# Supplementary material for: Differential nucleosome organization in human interphase and metaphase chromosomes
Source: Mol Syst Biol. 2026 Feb 2;22(5):738–65. doi: 10.1038/s44320-026-00192-y (PMC13144420; doi:10.1038/s44320-026-00192-y)
Supplement: Supplementary file 1 — Appendix [file 44320_2026_192_MOESM1_ESM.pdf]

Appendix PDF

Differential nucleosome organization in human interphase and metaphase chromosomes

Keren Li,[1,3,4,6] Irem Unlu,[2,6] Yiren Tu,[2,6] Lilien N. Voong,[2] Yanyan Lu,[2,3] Brody Kendall,[1] Xiaotian Ma,[1] Sin Lei Pui,[2] Meng Tao,[2] Ji-Ping Wang,[1,3,5,*] and Xiaozhong Wang, [2,3,5,*]

Table of contents

2 Appendix Table S1
3 Appendix Table S2, Appendix Table S3
4 Appendix Figure S1
5 Appendix Figure S2
6 Appendix Figure S3
7 Appendix Figure S4
8 Appendix Figure S5
9 Appendix Figure S6
10 Appendix Figure S7
11 Appendix Figure S8
12 Appendix Figure S9

Appendix Table S1: Correlation summaries within each Chromatin State

		Corr⩽0.15	Corr>0.15	odds	odds ratio	Percentage	Function	p-value
E1	ROI	3783	12238	30.91%	6.546	0.58%	Active TSS	0
	BG	123353	2612263	4.72%				
E2	ROI	1018	4802	21.20%	4.404	0.21%	Flanking TSS	1E-289
	BG	126118	2619699	4.81%				
E3	ROI	4794	12676	37.82%	8.074	0.64%	Flanking TSS Upstream	0
	BG	122342	2611825	4.68%				
E4	ROI	1944	7262	26.77%	5.596	0.33%	Flanking TSS Downstream	0
	BG	125192	2617239	4.78%				
E5	ROI	2723	69266	3.93%	0.807	2.62%	Strong transcription	5.69E-29
	BG	124413	2555235	4.87%				
E6	ROI	14530	314524	4.62%	0.948	11.96%	Weak transcription	2.07E-09
	BG	112606	2309977	4.87%				
E7	ROI	472	6560	7.20%	1.487	0.26%	Genic enhancer1	3.44E-15
	BG	126664	2617941	4.84%				
E8	ROI	265	2136	12.41%	2.564	0.09%	Genic enhancer2	1.34E-37
	BG	126871	2622365	4.84%				
E9	ROI	4359	17429	25.01%	5.311	0.79%	Active Enhancer 1	0
	BG	122777	2607072	4.71%				
E10	ROI	628	4807	13.06%	2.705	0.20%	Active Enhancer 2	6.01E-95
	BG	126508	2619694	4.83%				
E11	ROI	7461	52741	14.15%	3.040	2.19%	Weak Enhancer	0
	BG	119675	2571760	4.65%				
E12	ROI	156	3073	5.08%	1.048	0.12%	ZNF genes & repeats	0.557024
	BG	126980	2621428	4.84%				
E13	ROI	1020	38312	2.66%	0.546	1.43%	Heterochromatin	3.14E-97
	BG	126116	2586189	4.88%				
E14	ROI	29	293	9.90%	2.043	0.01%	Bivalent/Poised TSS	0.000735
	BG	127107	2624208	4.84%				
E15	ROI	18	265	6.79%	1.402	0.01%	Bivalent Enhancer	0.15662
	BG	127118	2624236	4.84%				
E16	ROI	1317	38009	3.46%	0.712	1.43%	Repressed PolyComb	8.42E-37
	BG	125819	2586492	4.86%				
E17	ROI	13046	335263	3.89%	0.781	12.66%	Weak Repressed PolyComb	3.6E-161
	BG	114090	2289238	4.98%				
E18	ROI	69573	1704845	4.08%	0.652	64.49%	Quiescent/Low	0
	BG	57563	919656	6.26%				

Appendix Table S2: Linker Length (l) Comparisons Among Chromatin States

	Interphase		Metaphase		odd ratio	Test p-value		
	$\#(l \leqslant 30)$	$\#(l > 30)$	$\#(l \leqslant 30)$	$\#(l > 30)$		vs E5-E6	vs E13	vs E18
E1-E4	57230	87033	40225	104294	1.705	0	0	0
E5-E6	582841	960381	550112	1075258	1.186	--	--	--
E9-E11	108386	166771	82769	200619	1.575	0	0	0
E13	56260	96007	55364	101836	1.078	--	--	--
E18	2341812	4613845	2147515	5134842	1.214	--	--	--

All comparisons yielded highly significant results (p-values ≈ 0), indicating that interphase enrichment was significantly stronger in E1–E4 and E9–E11 compared to E5–E6, E13, or E18. The odds ratios were 1.705 for E1–E4 and 1.575 for E9–E11, substantially higher than those observed for E5–E6 (1.186), E13 (1.078), and E18 (1.214). These findings demonstrate that E1–E4 and E9–E11 exhibit markedly greater enrichment gains during the transition from metaphase to interphase.

Appendix Table S3: Linker Length (l) Comparisons for TSS, Active Enhancer, and CTCF

	Interphase		Metaphase		odd ratio	Test vs rest Whole Genome region	
	$\#(l \leqslant 30)$	$\#(l > 30)$	$\#(l \leqslant 30)$	$\#(l > 30)$		odd ratio (other region)	p-value
TSS	48453	79791	36025	93094	1.569	1.225	0
Active Enhancer	26854	39759	19893	49023	1.664	1.226	0
CTCF	219217	305247	153377	379739	1.778	1.205	0
Whole	3716677	7232156	3374368	8063849	1.228	--	--

All comparisons yielded highly significant results (p-values ≈ 0), indicating that short linker length enrichment in interphase was significantly stronger in TSS, active enhancer, and CTCF regions compared to genome-wide background regions with those elements excluded. Specifically, the odds ratios for interphase over metaphase were 1.569 for TSSs, 1.664 for active enhancers, and 1.778 for CTCF sites, each notably higher than the corresponding background odds ratios of 1.225, 1.226, and 1.205 (1.228 for the whole genome). These findings demonstrate that these regulatory regions exhibit markedly greater gains in short linker length during the transition from metaphase to interphase compared to the rest of the genome.

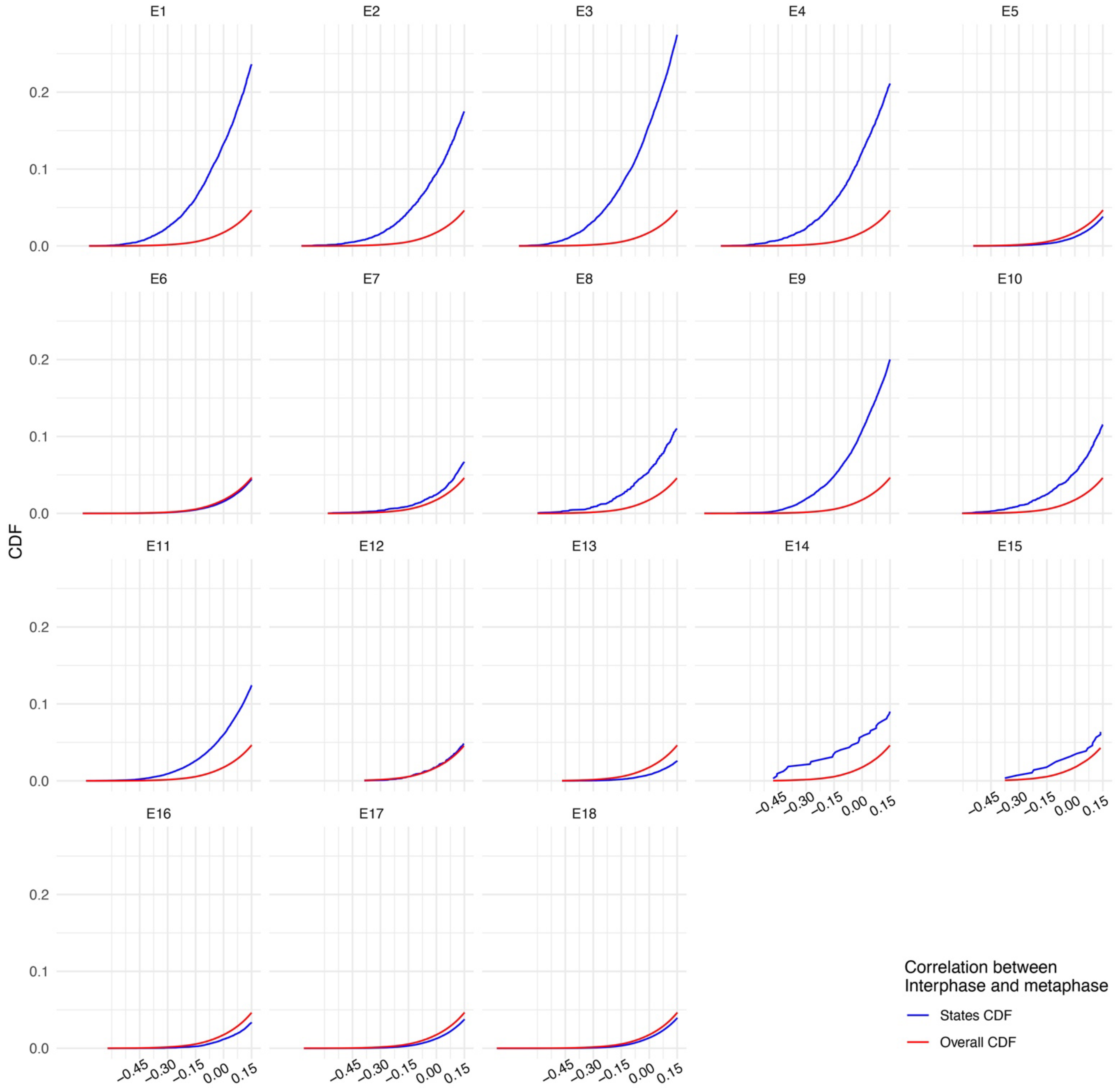

Appendix Figure S1. ECDF plots of local nucleosome occupancy correlation between interphase and metaphase across 18 chromatin states. Empirical cumulative distribution function (ECDF) curves illustrate the local correlation in nucleosome occupancy between interphase and metaphase across 18 chromatin states defined by ChromHMM in HeLa S3 cells. ECDF curves for chromatin states E1, E2, E3, E4, E8, E9, E10, and E11 lie well above the genome-wide curve, indicating a marked enrichment of regions with low interphase–metaphase correlation.

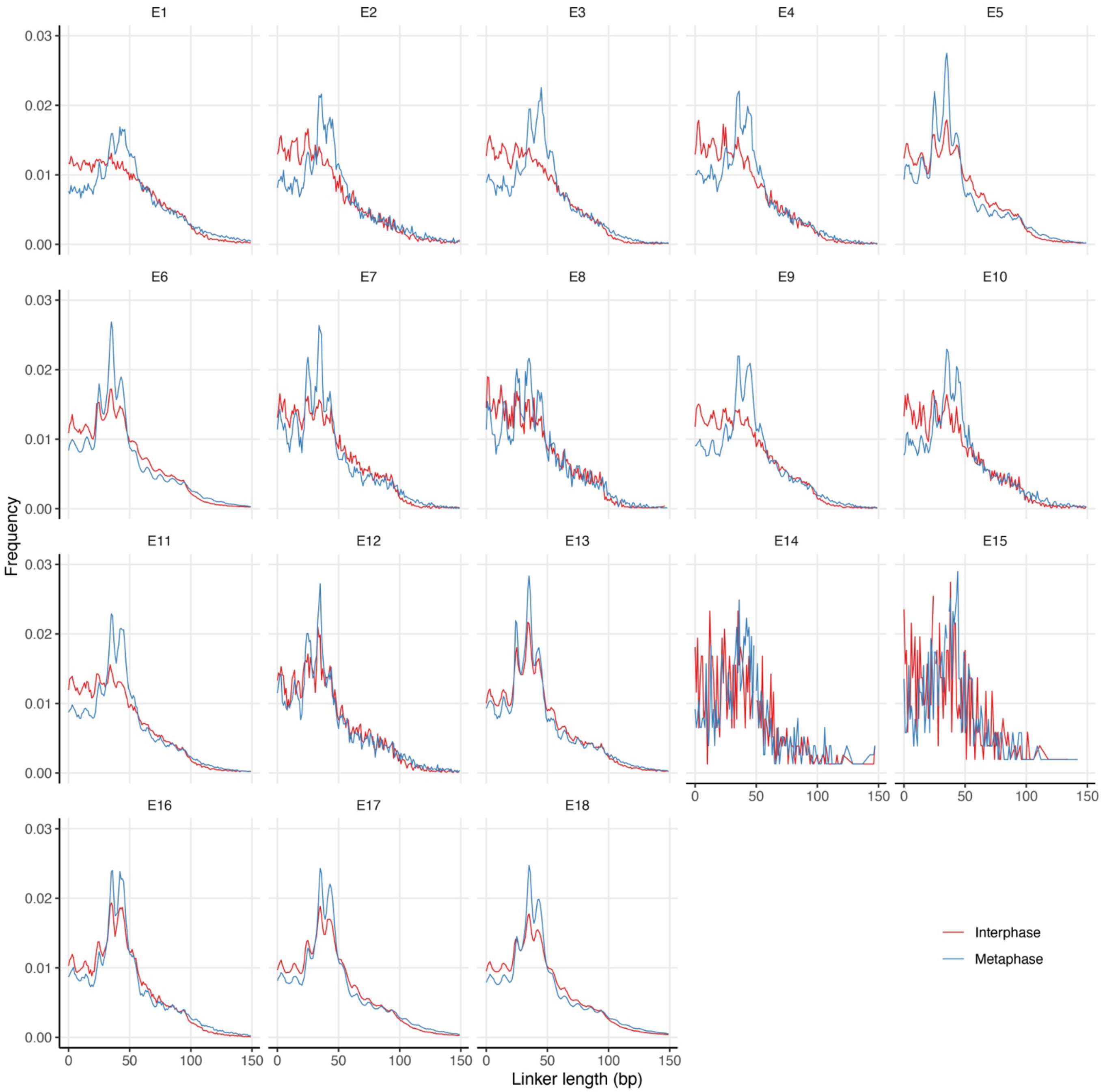

Appendix Figure S2. Linker DNA length distribution in interphase and metaphase across chromatin states. Distribution of linker lengths inferred from unique nucleosome maps in interphase and metaphase across 18 chromatin states defined by ChromHMM in HeLa S3 cells.

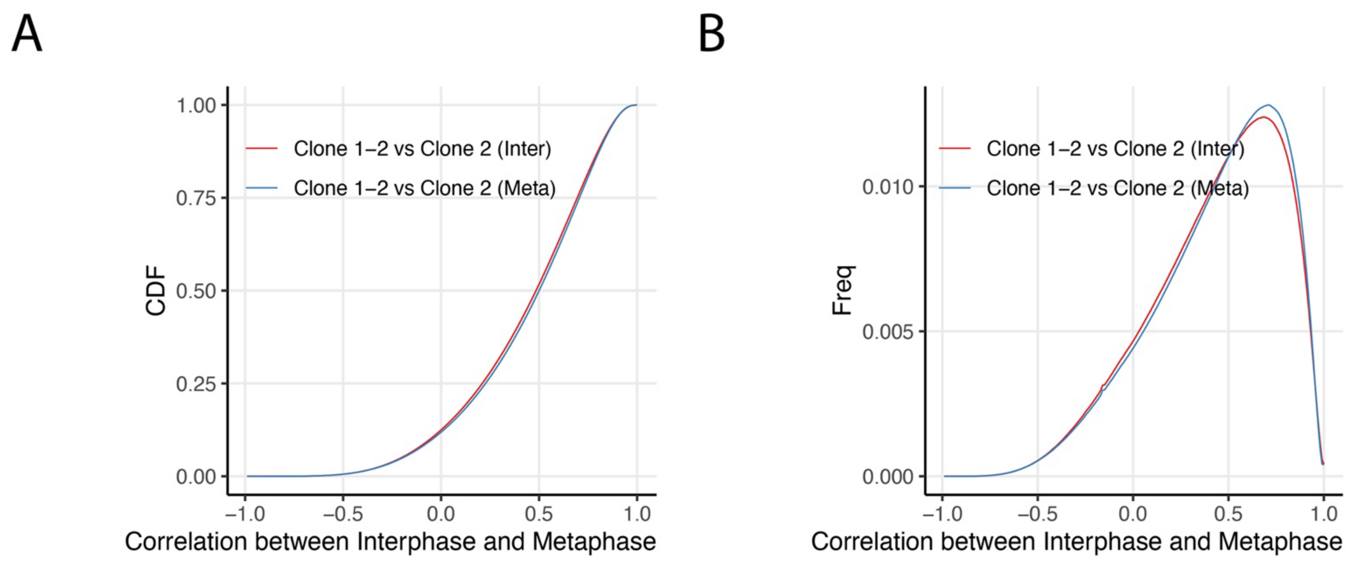

Appendix Figure S3. Genome-wide comparison of interphase and metaphase chemical nucleosome maps from clone-2 and clone 1-2. (**A**) Empirical cumulative distribution function (ECDF) plots showing genome-wide local Pearson correlation of interphase and metaphase nucleosome occupancy scores between two H4S47C-expressing cell lines, clone-2 and clone 1-2. Correlations were calculated using a 501-bp sliding window with a 1-bp step size. (**B**) Frequency distribution of local correlation values for the same comparisons shown in (A).

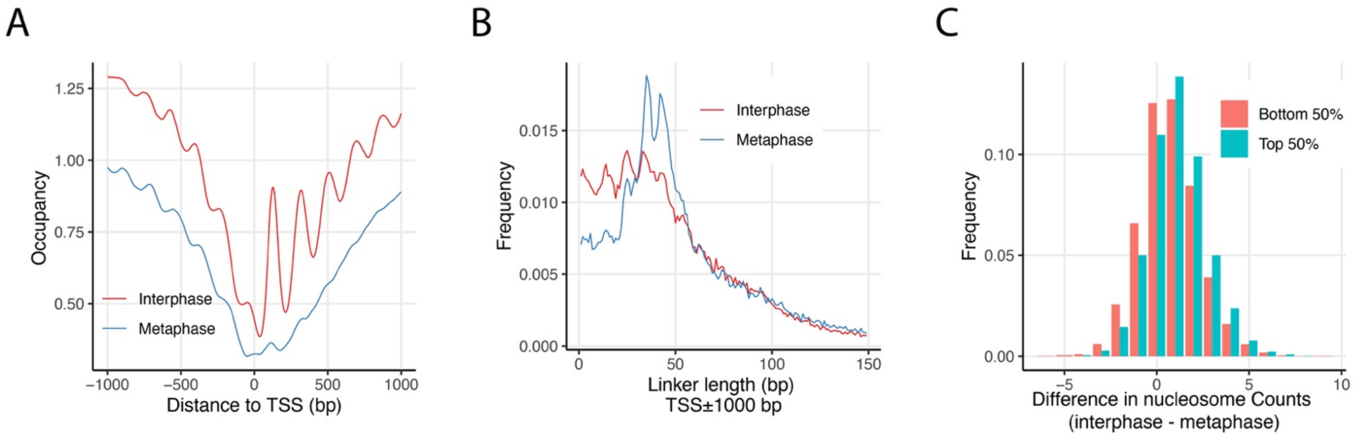

Appendix Figure S4. Features of nucleosome positioning around active promoters in clone 1-2. (**A**) Plot of interphase and metaphase nucleosome occupancy scores, averaged at the TSSs of 15,073 expressed protein genes. (**B**) Linker length distributions near TSSs showing that shorter linkers are further enriched in promoter regions during interphase. (**C**) Histogram showing the difference in unique nucleosome counts within ±1000 bp of each TSS between interphase and metaphase, for genes in the top and bottom 50% of expression levels.

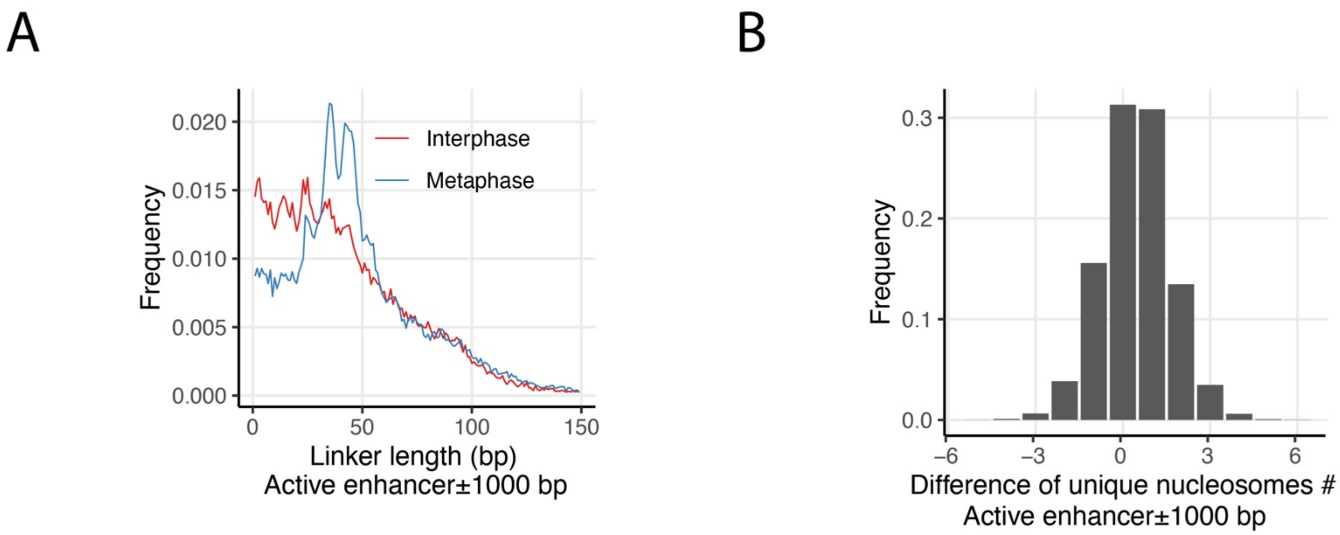

Appendix Figure S5. Nucleosome organization at active enhancers during mitosis in clone 1-2. (**A**) Linker length distributions near active enhancers in interphase versus metaphase. (**B**) Histogram showing the difference in unique nucleosome counts within ±1000 bp of each active enhancer between interphase and metaphase. On average, interphase exhibits 0.455 more unique nucleosomes per enhancer than metaphase.

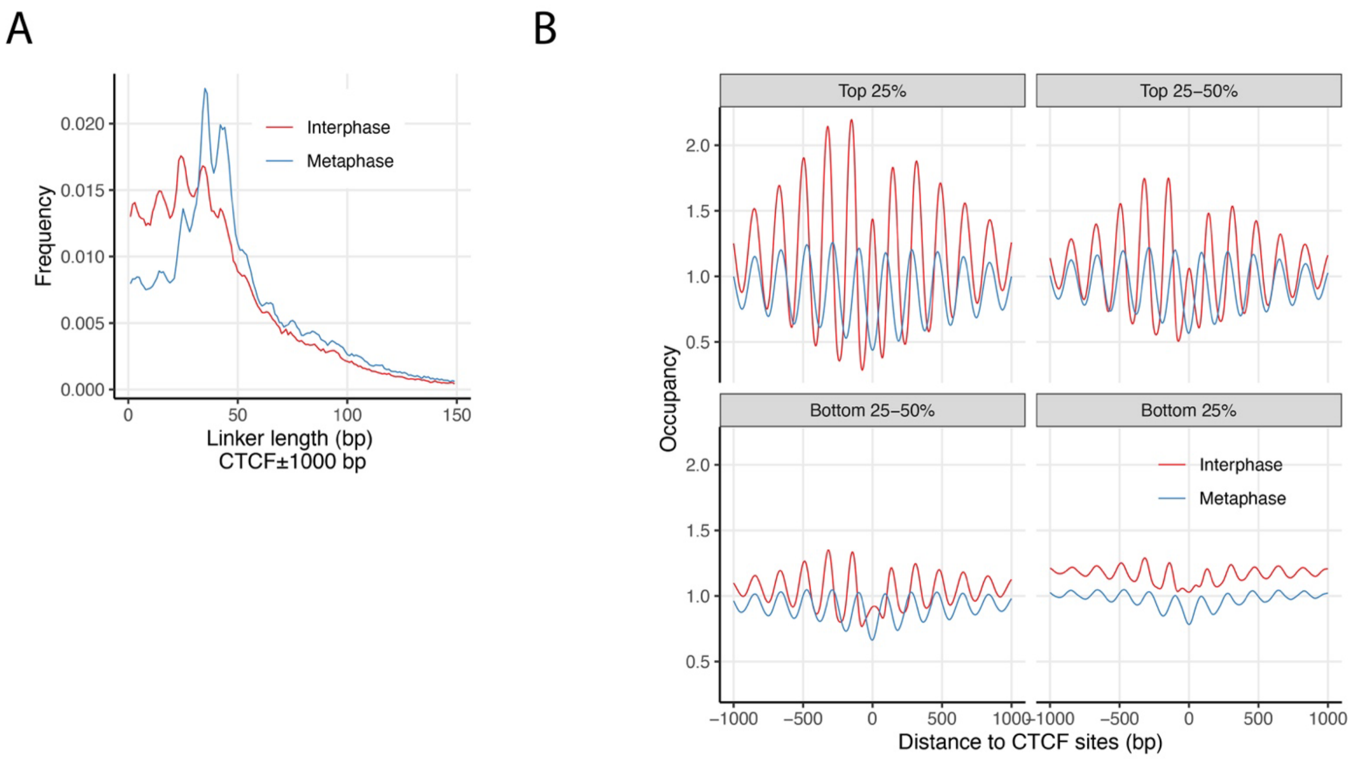

Appendix Figure S6. Nucleosome positioning at CTCF binding sites during mitosis in clone 1-2. (**A**) Comparison of linker length distributions near CTCF binding sites in interphase and metaphase genomes. (**B**) Nucleosome occupancy scores at CTCF binding sites from interphase and metaphase chemical maps, with nucleosome occupancy scores further divided into quartiles based on CTCF ChIP-seq signals.

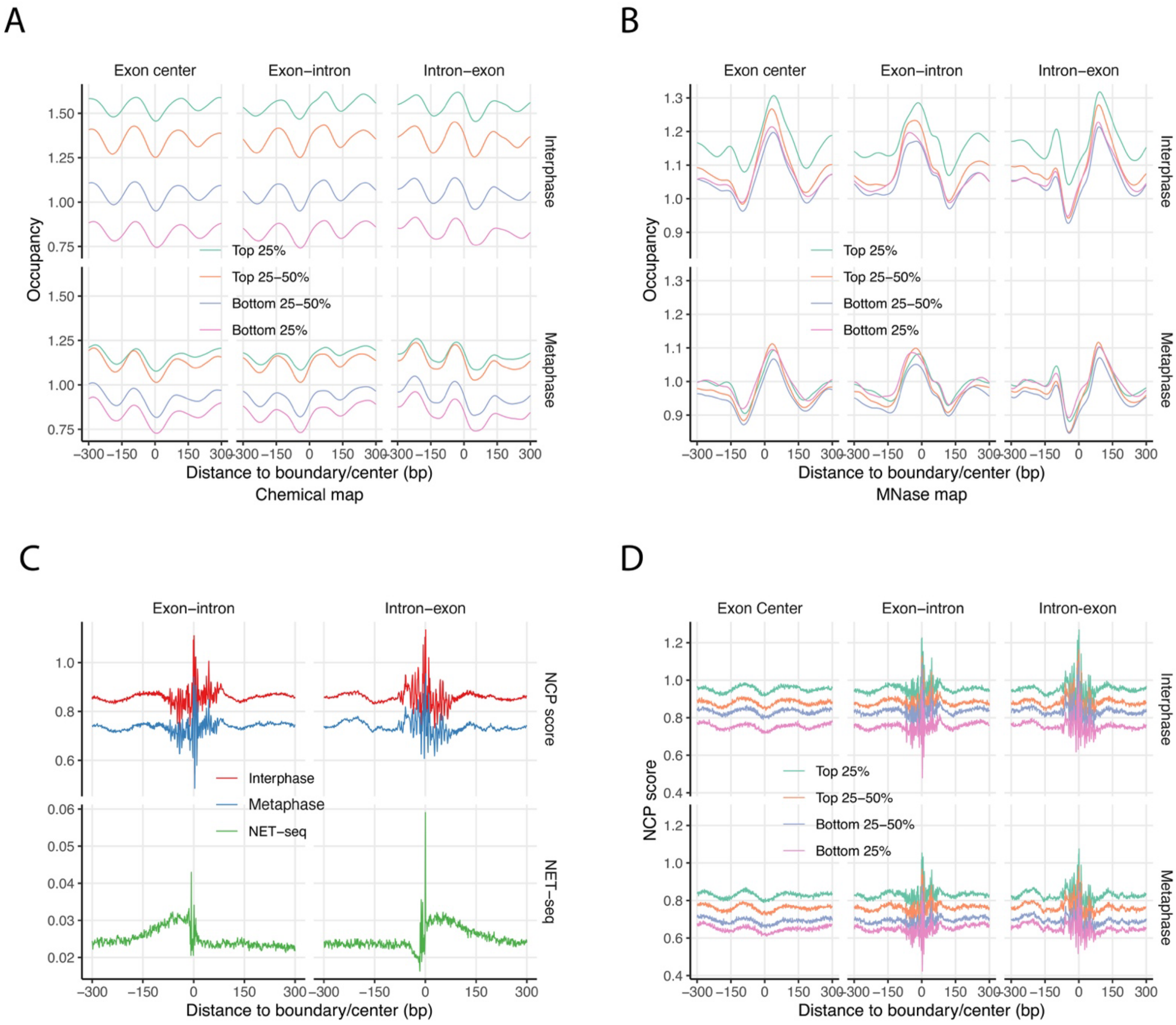

Appendix Figure S7. Nucleosome positioning at exon-intron and intron-exon junctions in clone 1-2. (**A**) Nucleosome occupancy scores from chemical maps of interphase (upper panels) and metaphase (lower panels), averaged at exon centers, exon-intron junctions, and intron-exon junctions. A total of 164,916 internal exons from 19,166 protein-coding genes were analyzed and grouped into quartiles based on gene expression levels (FPKM). 22,916 exons from non-expressed genes were excluded. (**B**) Same as panel (A) but using MNase-derived nucleosome maps. (**C**) NCP scores from interphase (red) and metaphase (blue) chemical maps at exon-intron and intron-exon junctions (upper panels), alongside NET-seq data averaged at the same positions (lower panels). (**D**) Chemical NCP scores from interphase (upper panels) and metaphase (lower panels) averaged at exon centers and junctions, stratified into expression-level quartiles based on gene FPKM values.

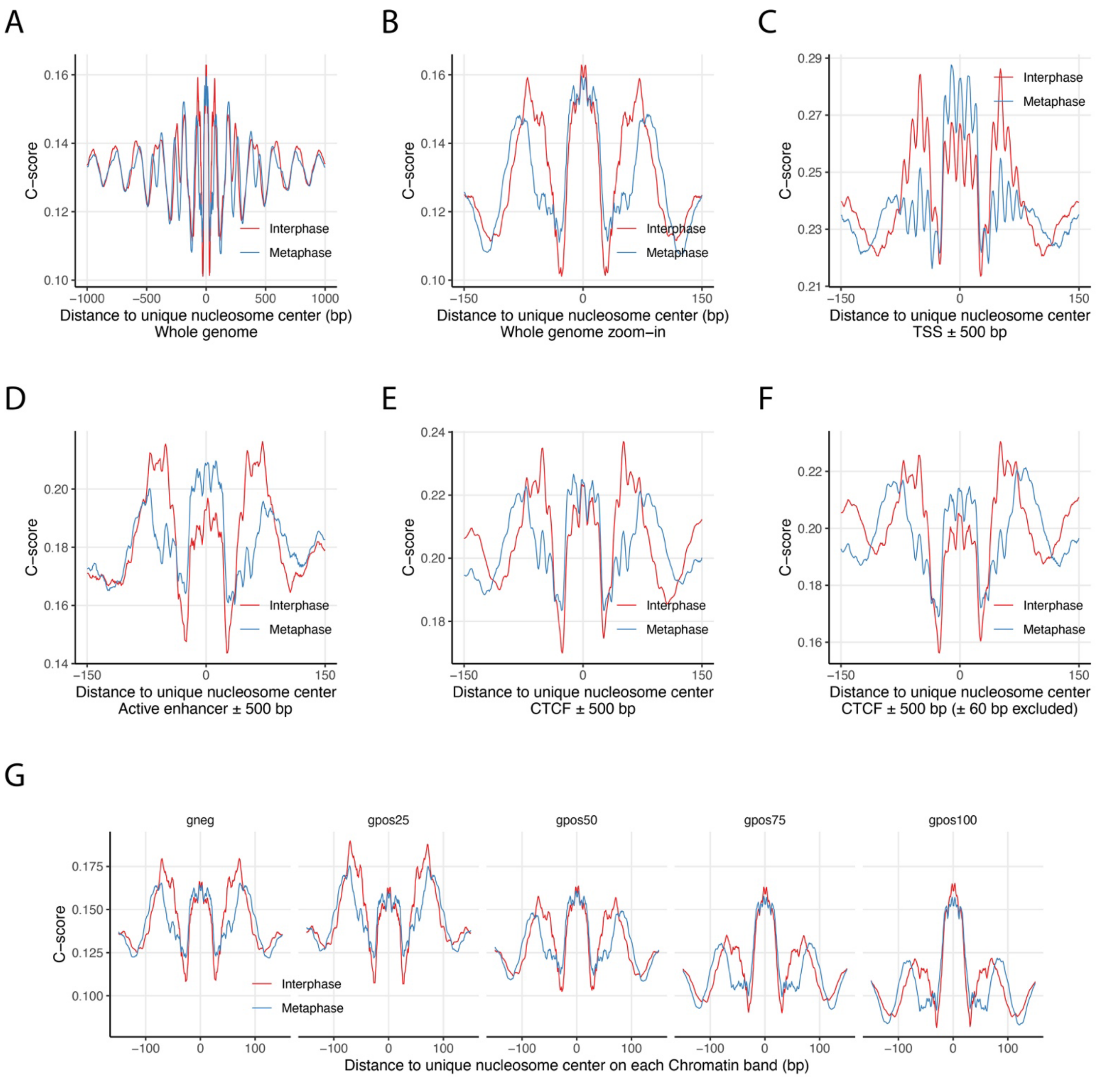

Appendix Figure S8. Chemical mapping in independent H4S47C-expressing HeLa S3 cells (clone 1-2) verifies the relationship between DNA cyclizability and nucleosome repositioning during mitosis. (**A**) Average predicted DNA cyclizability scores (C-scores) for unique nucleosomes and their flanking regions derived from genome-wide interphase and metaphase chemical maps in clone 1-2. (**B**) Zoomed-in view of average genome-wide C-scores within the nucleosome region, comparing interphase and metaphase, corresponding to (A). (**C, D**) Interphase and metaphase nucleosomal C-scores within ±500 bp of transcription start sites (C) and active enhancers (D). (**E**) Nucleosomal C-scores within ±500 bp of CTCF binding sites in interphase and metaphase. (**F**) Same as (E), but for unique nucleosomes located at least 60 bp from the CTCF motif center. (**G**) Average nucleosomal C-scores across chromosomal regions ranked by Giemsa staining patterns.

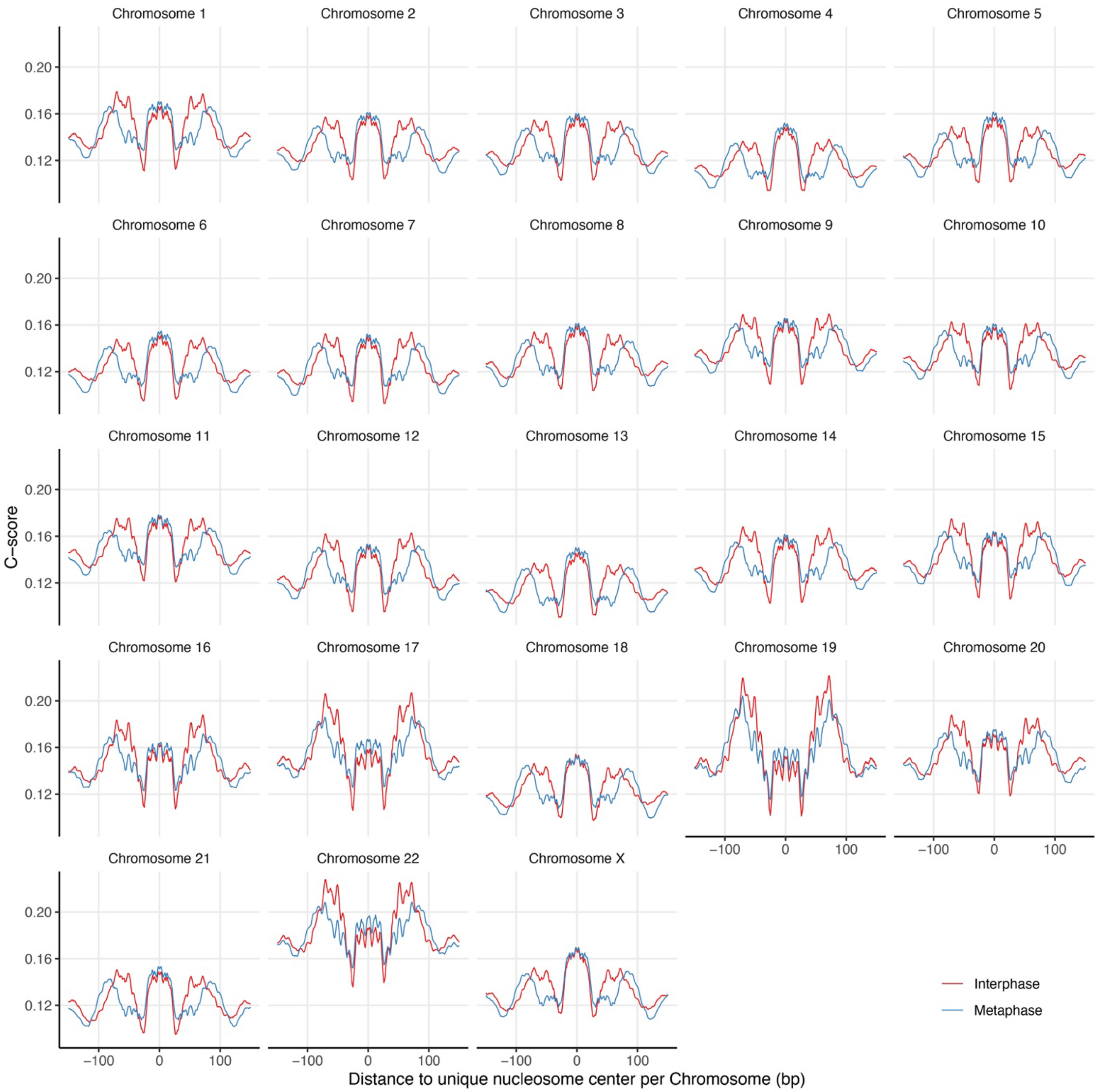

Appendix Figure S9. DNA cyclizability scores across individual human chromosomes. Comparison of nucleosomal C-scores between interphase and metaphase across all human chromosomes.
