# Supplementary figures and images for: Differential nucleosome organization in human interphase and metaphase chromosomes
Source: Mol Syst Biol. 2026 Feb 2;22(5):738–65. doi: 10.1038/s44320-026-00192-y (PMC13144420; doi:10.1038/s44320-026-00192-y)

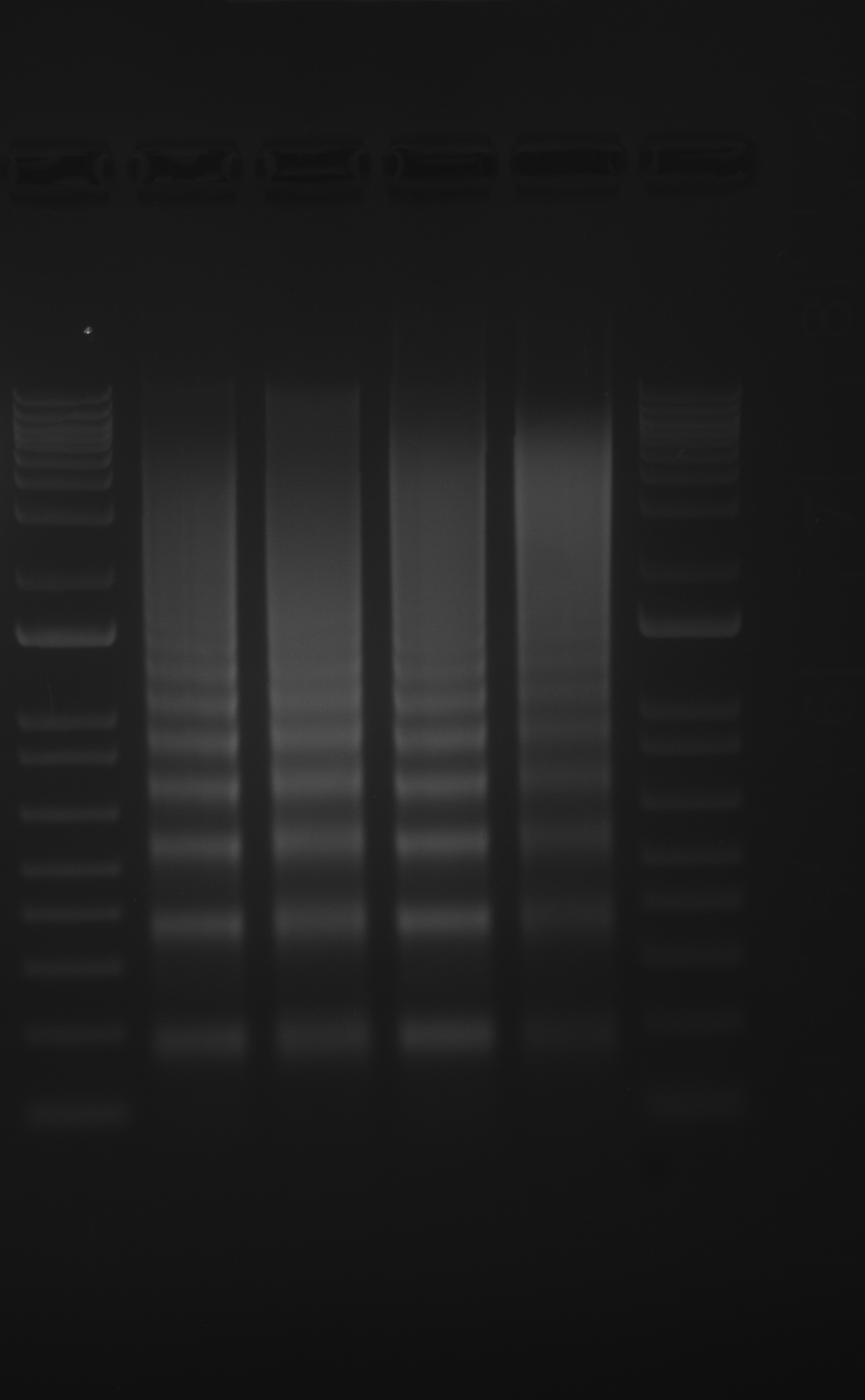

Supplement: Supplementary file 3 — Source data Fig. 1 [file 44320_2026_192_MOESM3_ESM.zip › Figure 1/1A/Fig1A.tif]

Cleavage Frequency

NS\_merge

M\_merge

0.06

0.04

0.02

0.00

-80

-40

0

40

80

Position

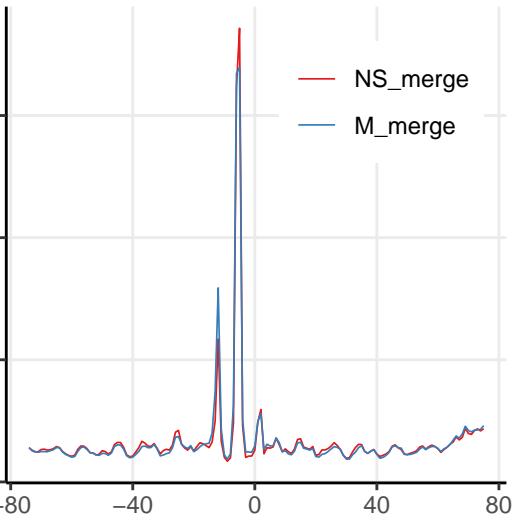

Supplement: Supplementary file 3 — Source data Fig. 1 [file 44320_2026_192_MOESM3_ESM.zip › Figure 1/1B/Figure1B.pdf]

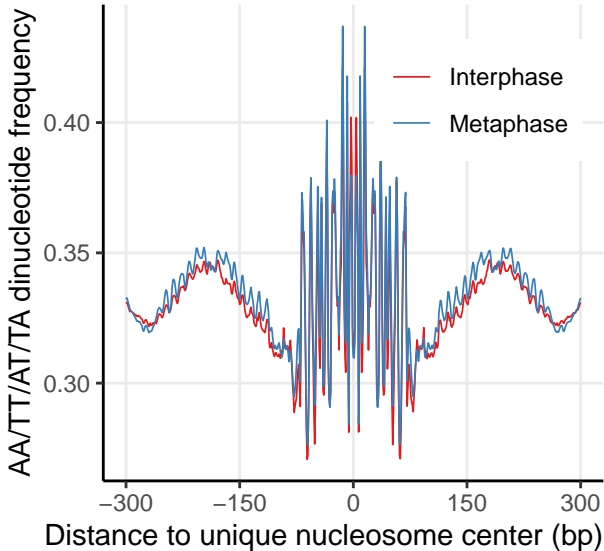

Supplement: Supplementary file 3 — Source data Fig. 1 [file 44320_2026_192_MOESM3_ESM.zip › Figure 1/1C/Figure1C.pdf]

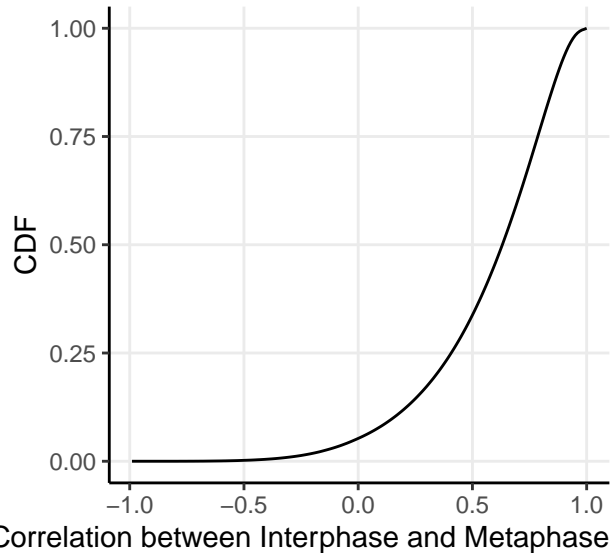

Supplement: Supplementary file 4 — Source data Fig. 2 [file 44320_2026_192_MOESM4_ESM.zip › Figure 2/2A/Figure2A.pdf]

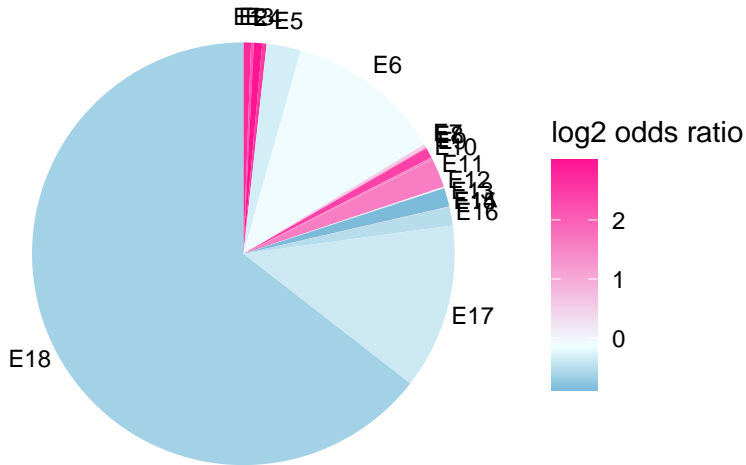

Supplement: Supplementary file 4 — Source data Fig. 2 [file 44320_2026_192_MOESM4_ESM.zip › Figure 2/2B/Figure2B.pdf]

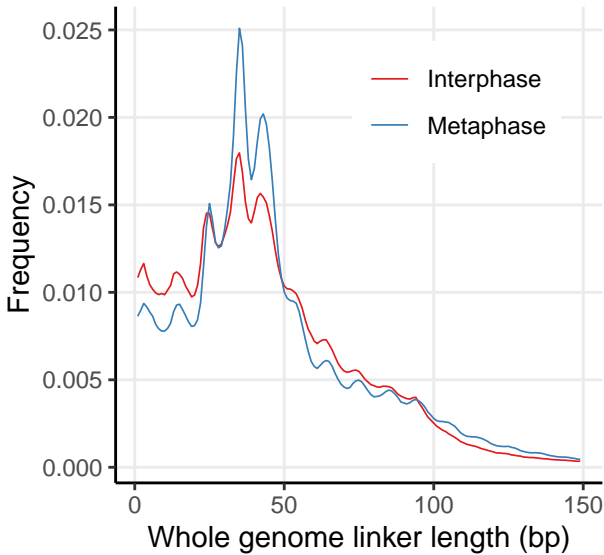

Supplement: Supplementary file 4 — Source data Fig. 2 [file 44320_2026_192_MOESM4_ESM.zip › Figure 2/2D/Figure2D.pdf]

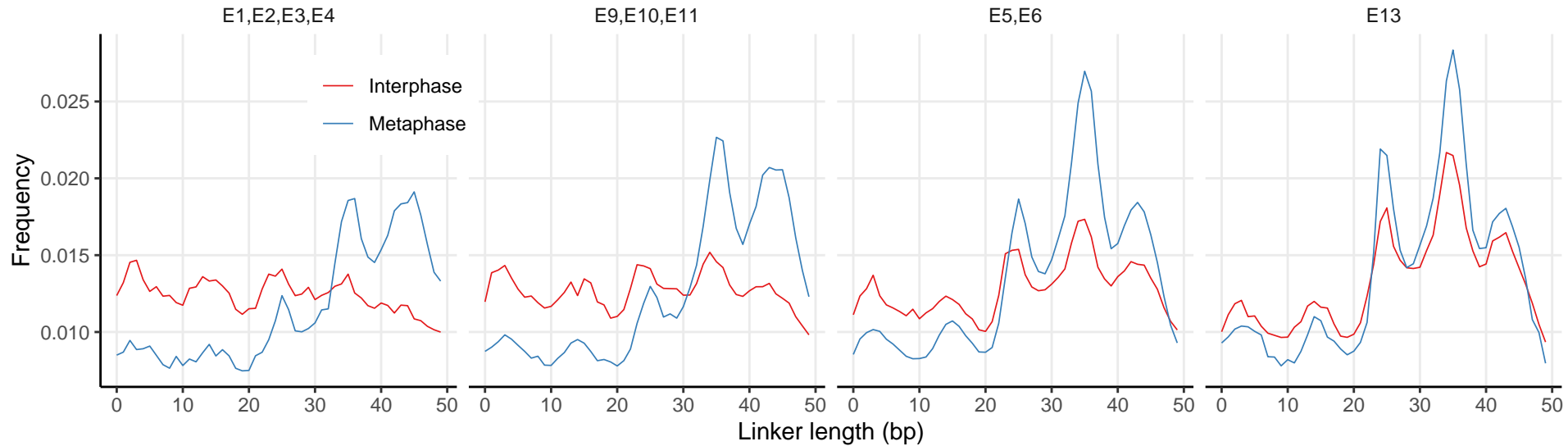

Supplement: Supplementary file 4 — Source data Fig. 2 [file 44320_2026_192_MOESM4_ESM.zip › Figure 2/2E/Figure2E.pdf]

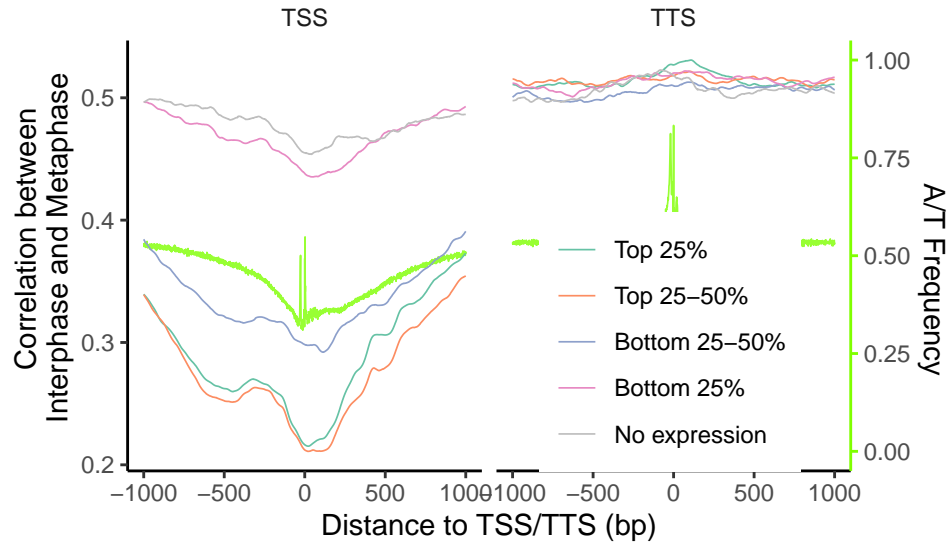

Supplement: Supplementary file 5 — Source data Fig. 3 [file 44320_2026_192_MOESM5_ESM.zip › Figure 3/3A/Figure3A.pdf]

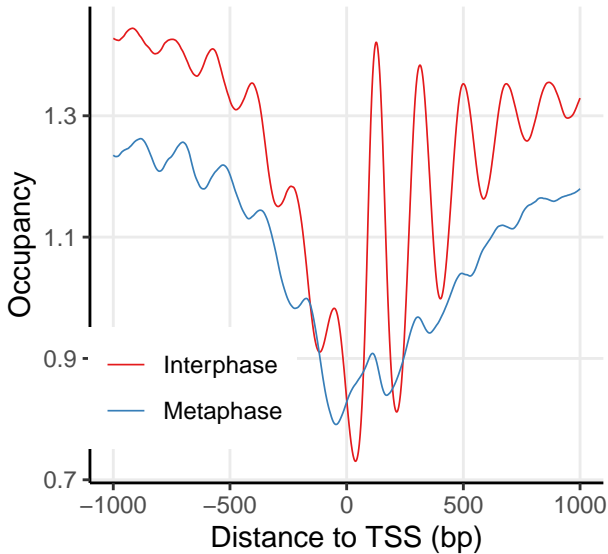

Supplement: Supplementary file 5 — Source data Fig. 3 [file 44320_2026_192_MOESM5_ESM.zip › Figure 3/3B/Figure3B.pdf]

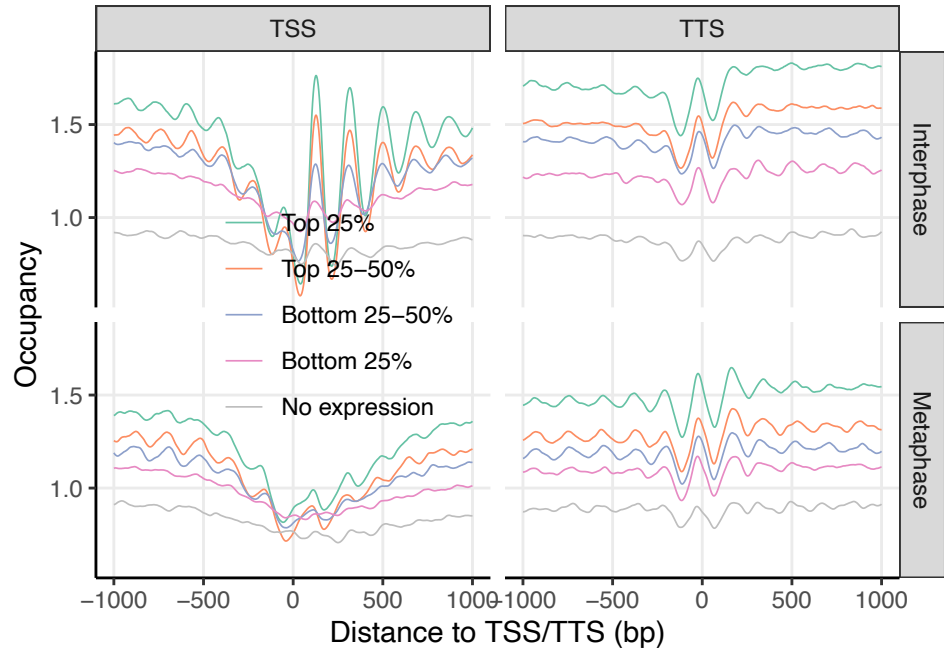

Supplement: Supplementary file 5 — Source data Fig. 3 [file 44320_2026_192_MOESM5_ESM.zip › Figure 3/3C/Figure3C.pdf]

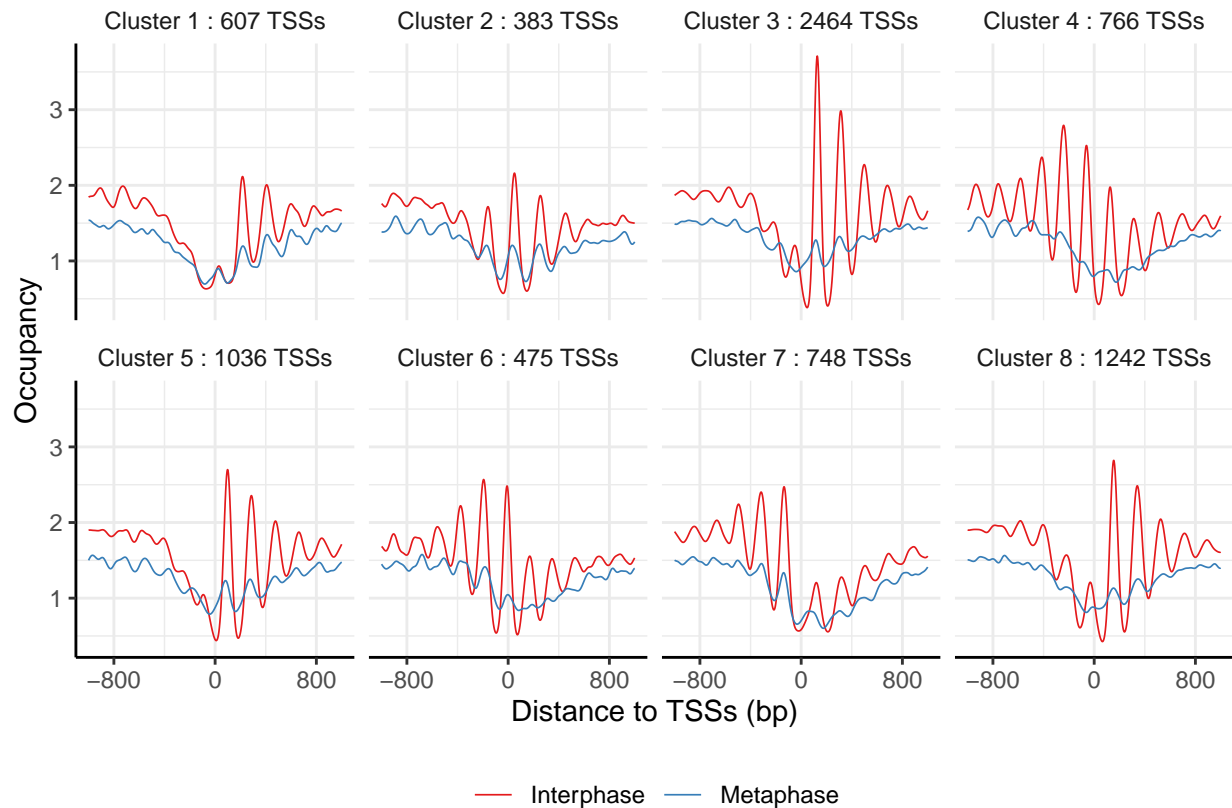

Supplement: Supplementary file 5 — Source data Fig. 3 [file 44320_2026_192_MOESM5_ESM.zip › Figure 3/3D/Figure3D.pdf]

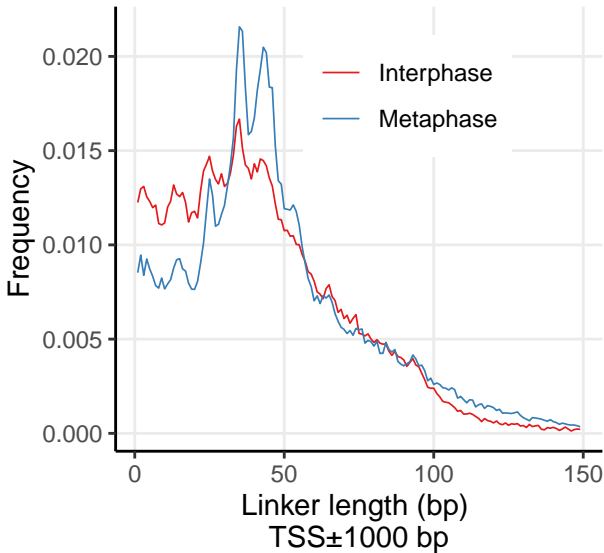

Supplement: Supplementary file 5 — Source data Fig. 3 [file 44320_2026_192_MOESM5_ESM.zip › Figure 3/3E/Figure3E.pdf]

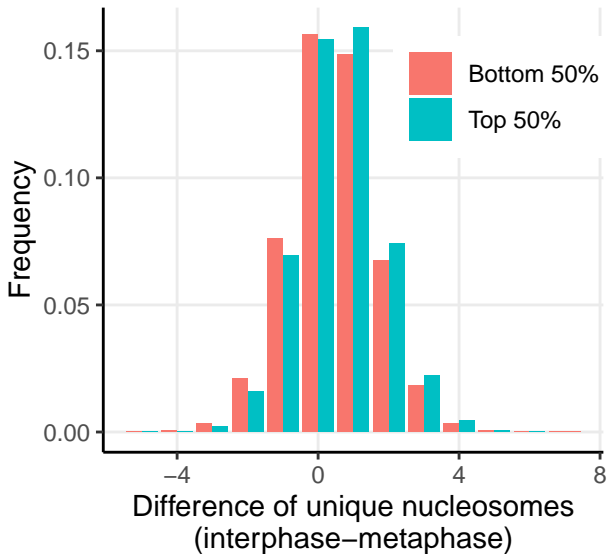

Supplement: Supplementary file 5 — Source data Fig. 3 [file 44320_2026_192_MOESM5_ESM.zip › Figure 3/3G/Figure3G.pdf]

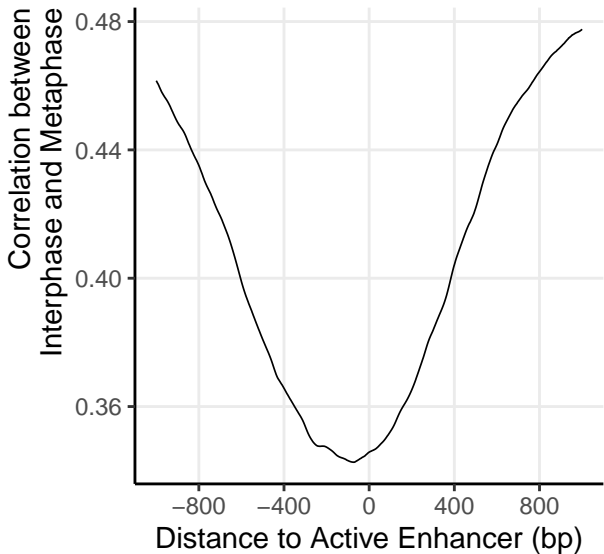

Supplement: Supplementary file 6 — Source data Fig. 4 [file 44320_2026_192_MOESM6_ESM.zip › Figure 4/4A/Figure4A.pdf]

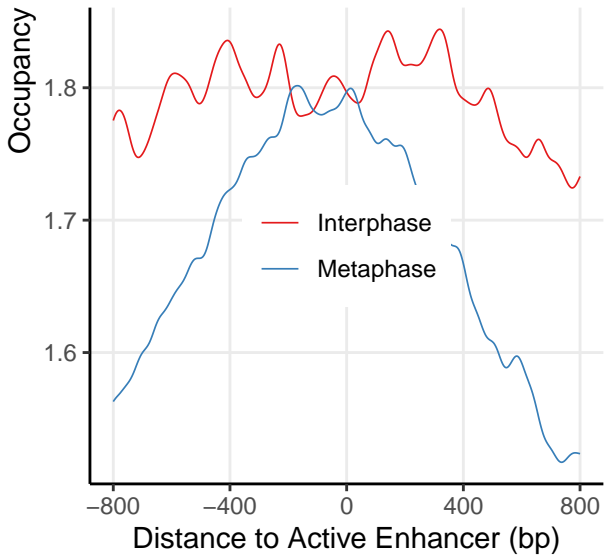

Supplement: Supplementary file 6 — Source data Fig. 4 [file 44320_2026_192_MOESM6_ESM.zip › Figure 4/4B/Figure4B.pdf]

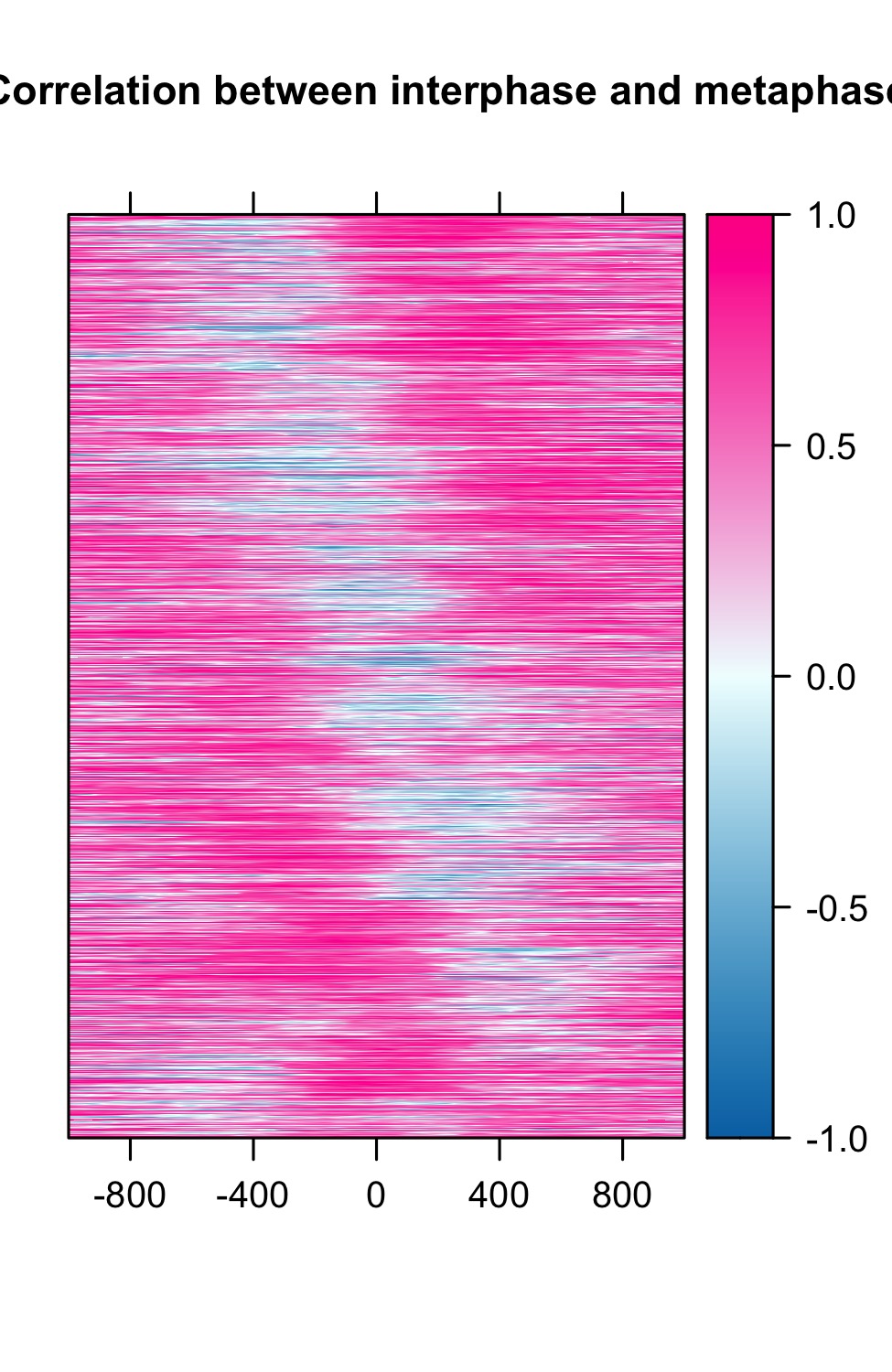

Supplement: Supplementary file 6 — Source data Fig. 4 [file 44320_2026_192_MOESM6_ESM.zip › Figure 4/4C/Figure4C.jpg]

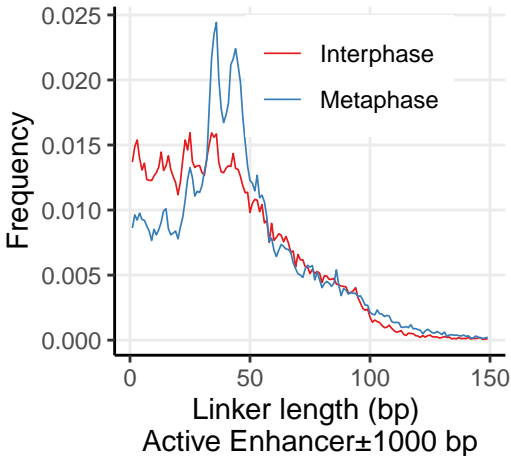

Supplement: Supplementary file 6 — Source data Fig. 4 [file 44320_2026_192_MOESM6_ESM.zip › Figure 4/4G/Figure4G.pdf]

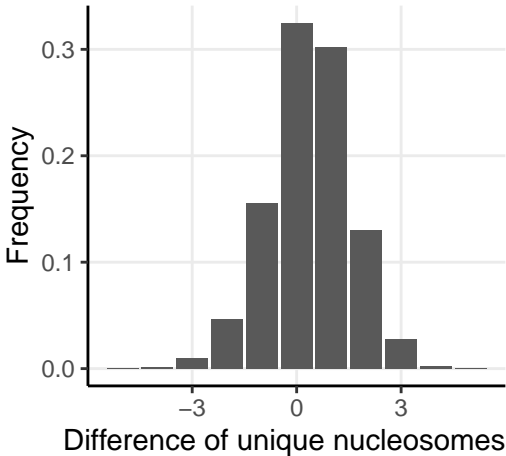

Supplement: Supplementary file 6 — Source data Fig. 4 [file 44320_2026_192_MOESM6_ESM.zip › Figure 4/4H/Figure4H.pdf]

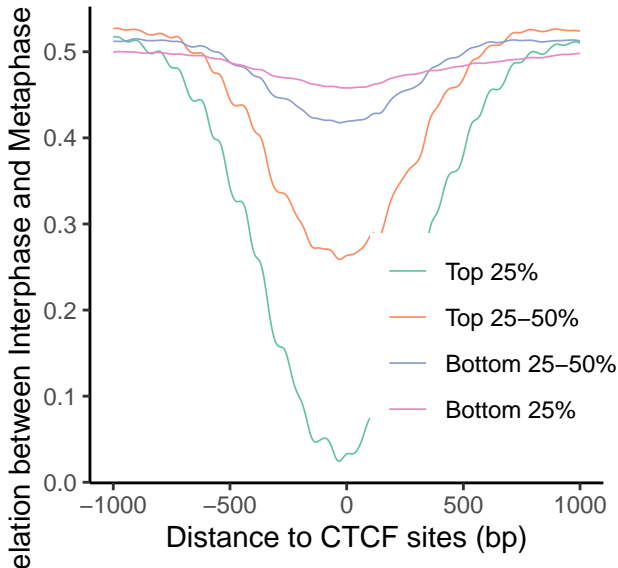

Supplement: Supplementary file 7 — Source data Fig. 5 [file 44320_2026_192_MOESM7_ESM.zip › Figure 5/5A/Figure5A.pdf]

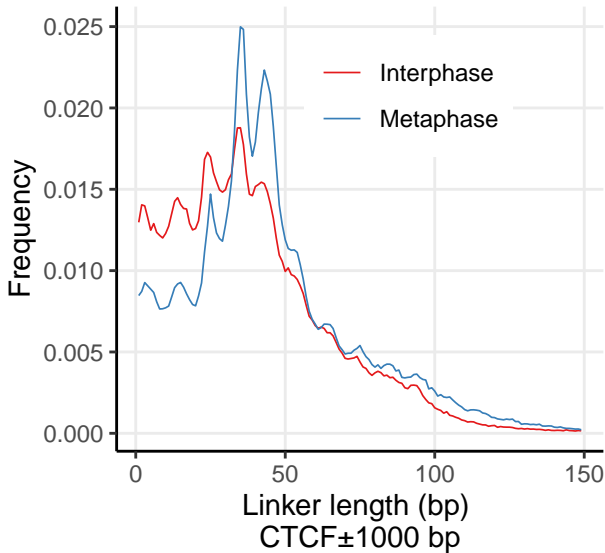

Supplement: Supplementary file 7 — Source data Fig. 5 [file 44320_2026_192_MOESM7_ESM.zip › Figure 5/5B/Figure5B.pdf]

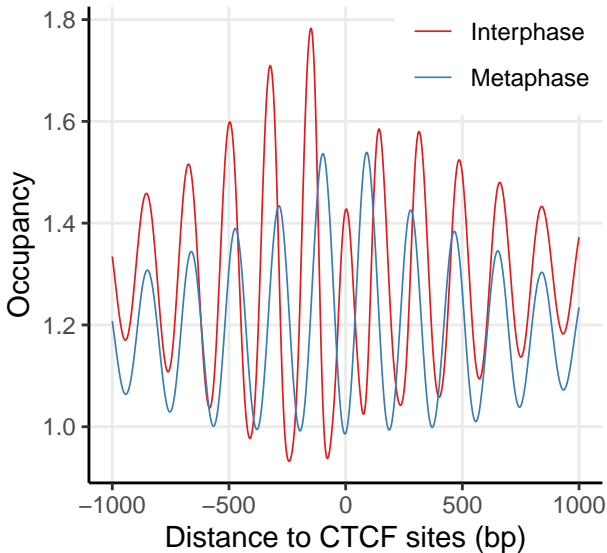

Supplement: Supplementary file 7 — Source data Fig. 5 [file 44320_2026_192_MOESM7_ESM.zip › Figure 5/5C/Figure5C.pdf]

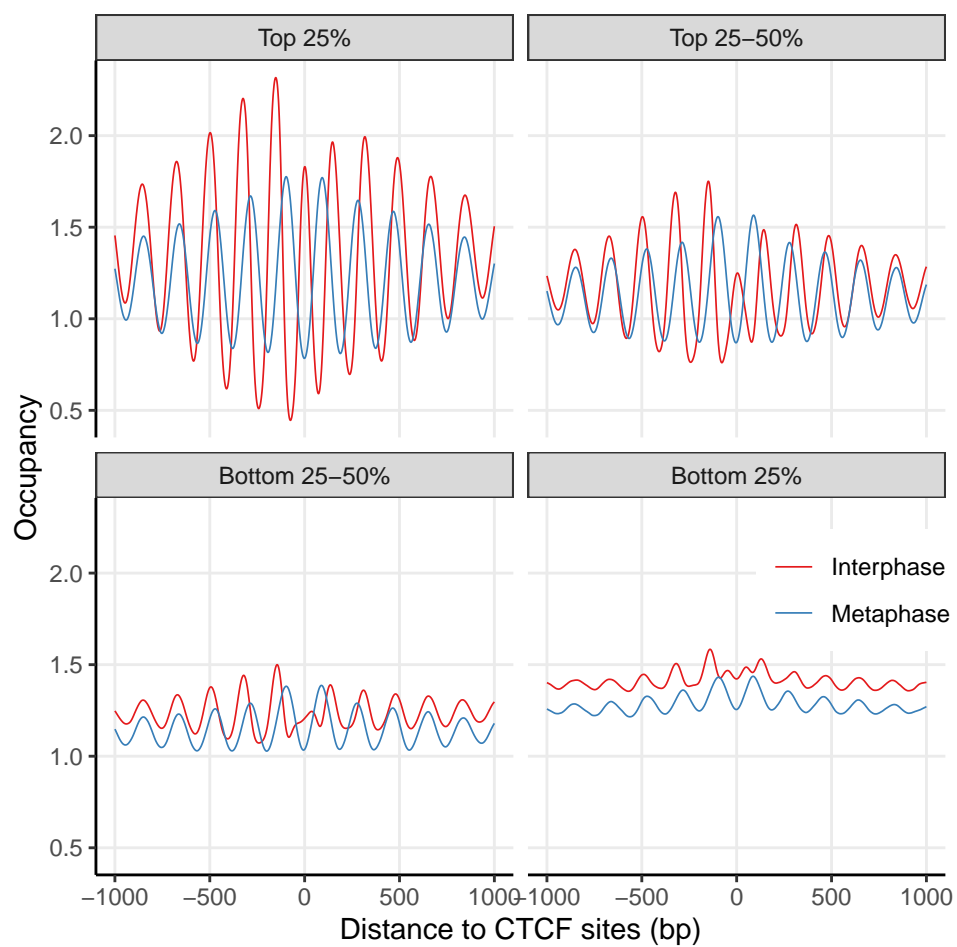

Supplement: Supplementary file 7 — Source data Fig. 5 [file 44320_2026_192_MOESM7_ESM.zip › Figure 5/5D/Figure5D.pdf]

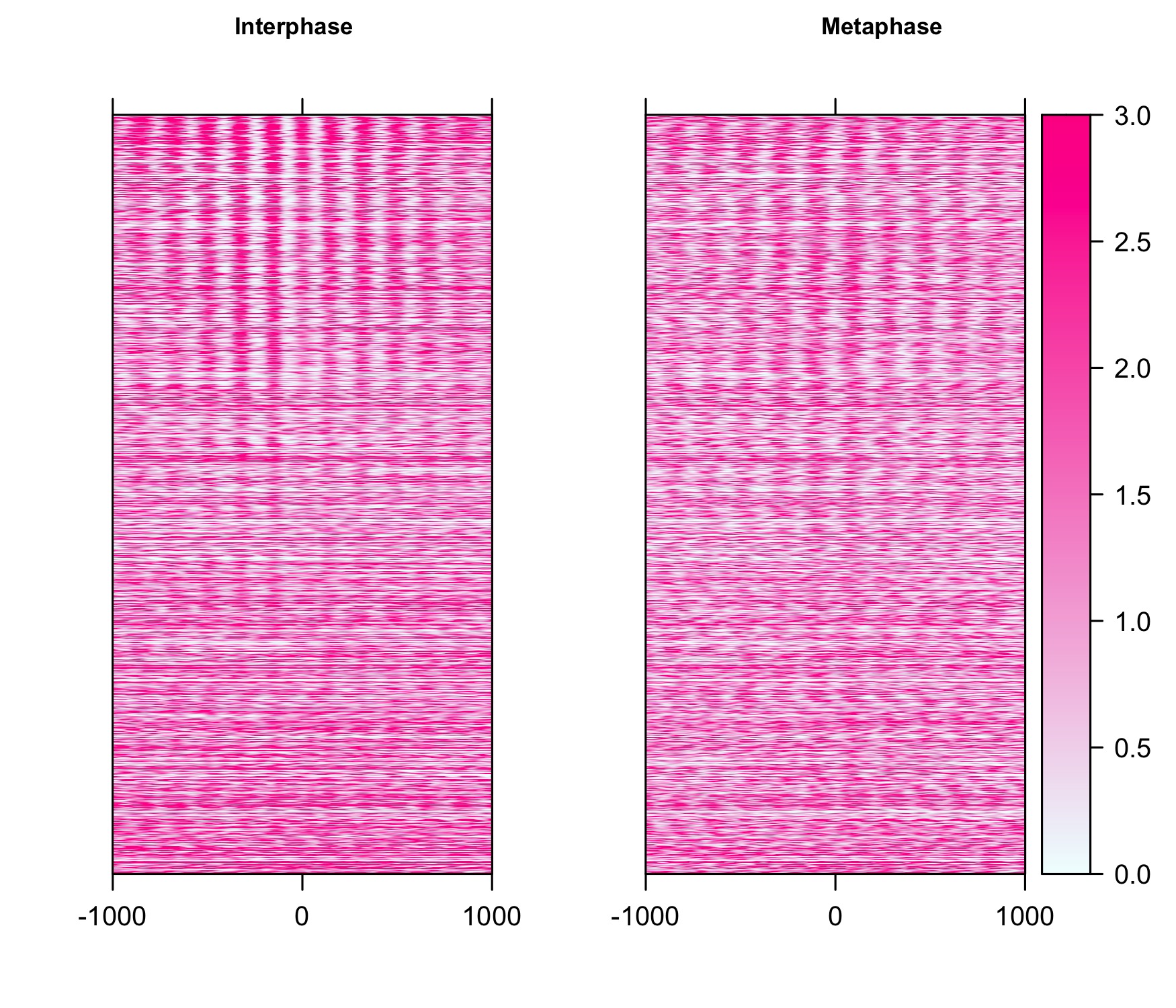

Supplement: Supplementary file 7 — Source data Fig. 5 [file 44320_2026_192_MOESM7_ESM.zip › Figure 5/5E/Figure5E.jpg]

— Top 25% — Top 25–50% — Bottom 25–50% — Bottom 25%

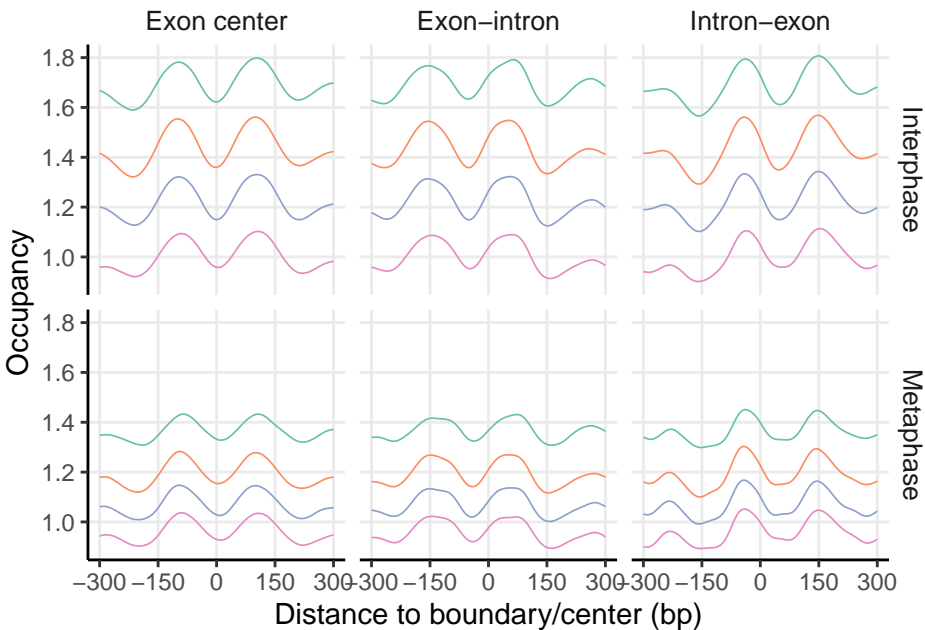

Supplement: Supplementary file 8 — Source data Fig. 6 [file 44320_2026_192_MOESM8_ESM.zip › Figure 6/6A/Figure6A.pdf]

Top 25%   Top 25–50%   Bottom 25–50%   Bottom 25%

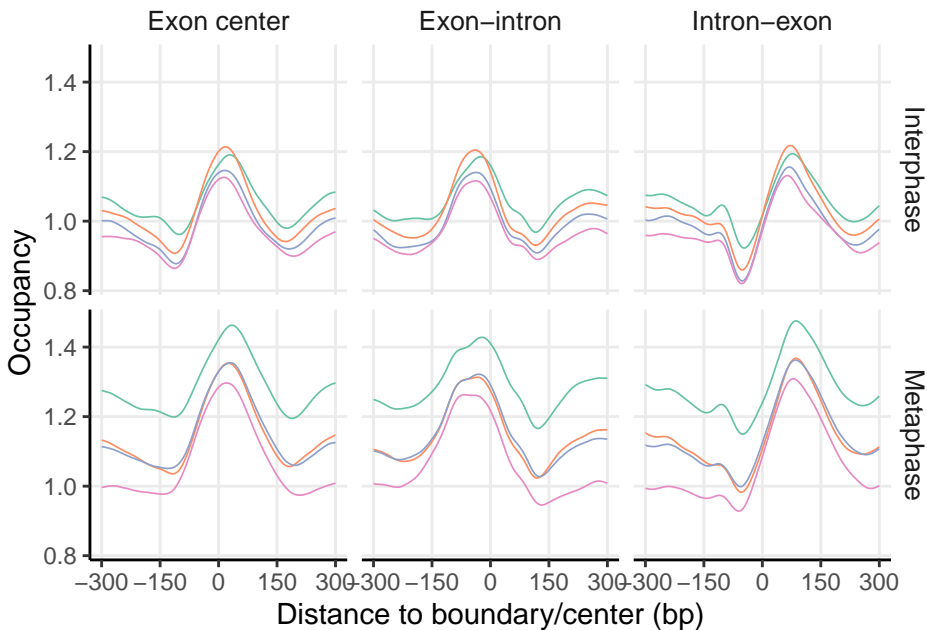

Supplement: Supplementary file 8 — Source data Fig. 6 [file 44320_2026_192_MOESM8_ESM.zip › Figure 6/6B/Figure6B.pdf]

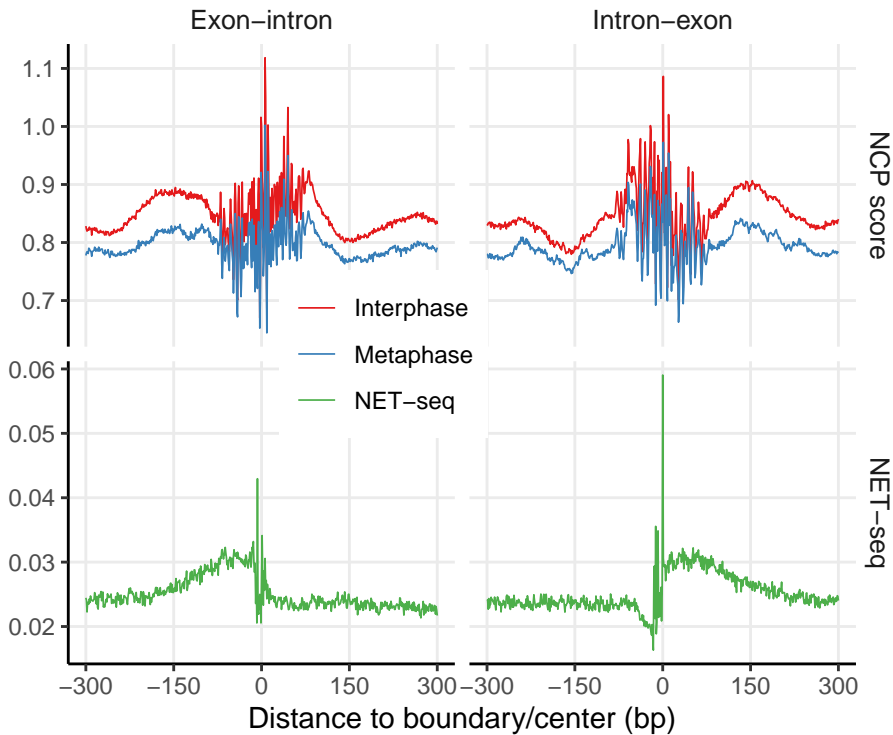

Supplement: Supplementary file 8 — Source data Fig. 6 [file 44320_2026_192_MOESM8_ESM.zip › Figure 6/6C/Figure6C.pdf]

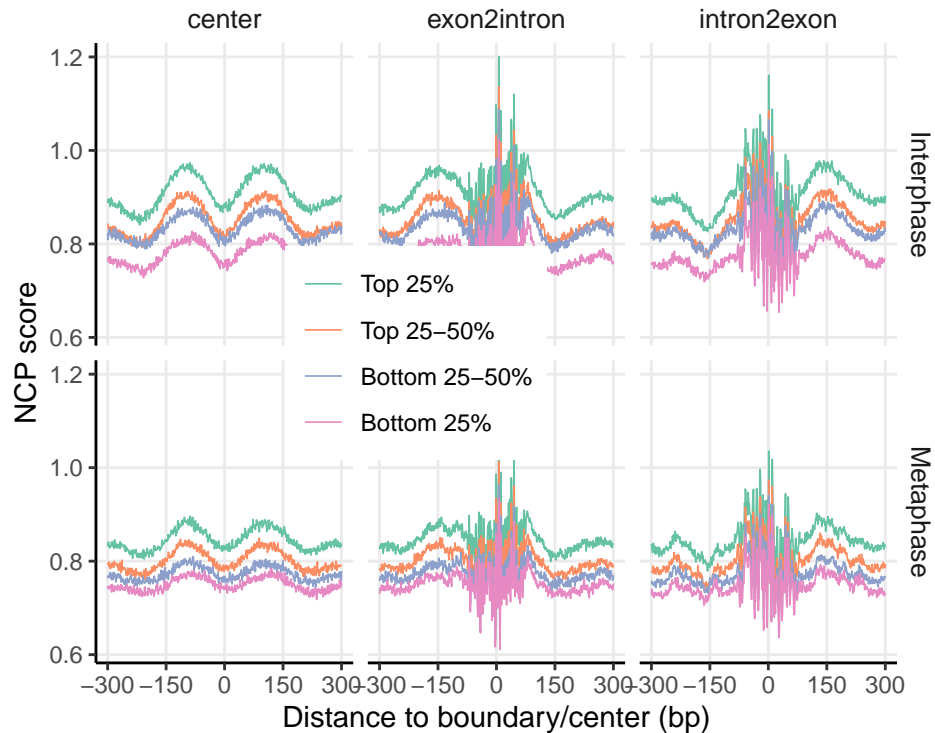

Supplement: Supplementary file 8 — Source data Fig. 6 [file 44320_2026_192_MOESM8_ESM.zip › Figure 6/6D/Figure6D.pdf]

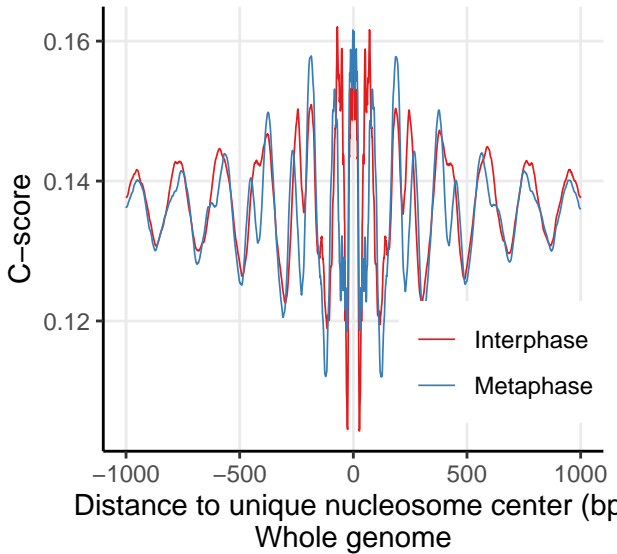

Supplement: Supplementary file 9 — Source data Fig. 7 [file 44320_2026_192_MOESM9_ESM.zip › Figure 7/7A/Figure7A.pdf]

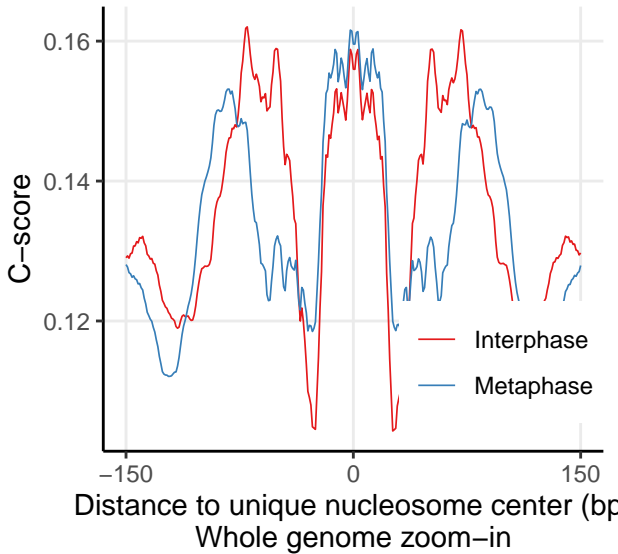

Supplement: Supplementary file 9 — Source data Fig. 7 [file 44320_2026_192_MOESM9_ESM.zip › Figure 7/7B/Figure7B.pdf]

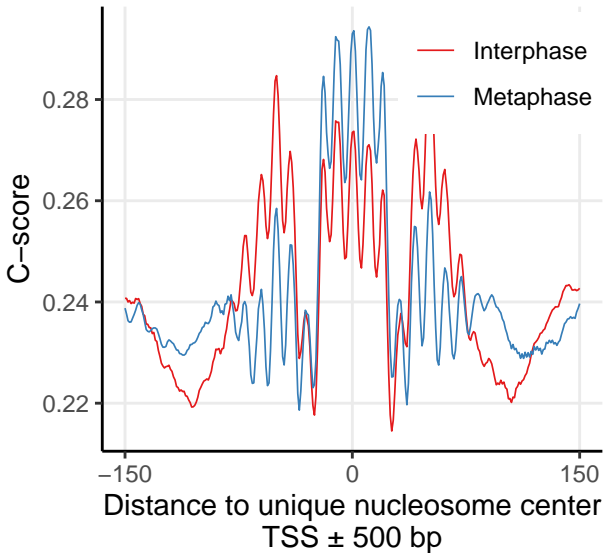

Supplement: Supplementary file 9 — Source data Fig. 7 [file 44320_2026_192_MOESM9_ESM.zip › Figure 7/7C/Figure7C.pdf]

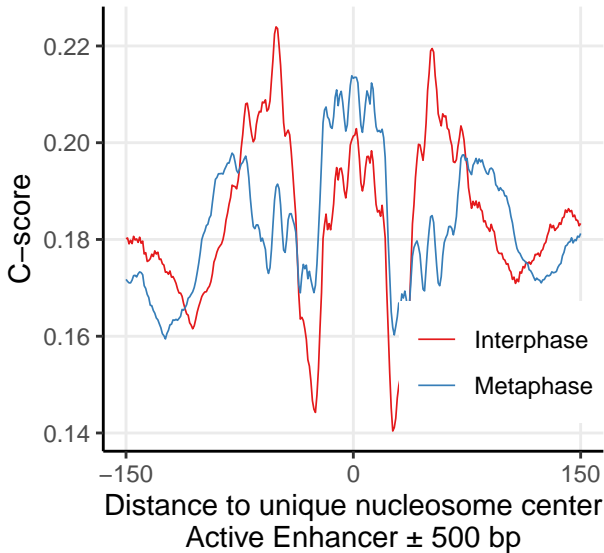

Supplement: Supplementary file 9 — Source data Fig. 7 [file 44320_2026_192_MOESM9_ESM.zip › Figure 7/7D/Figure7D.pdf]

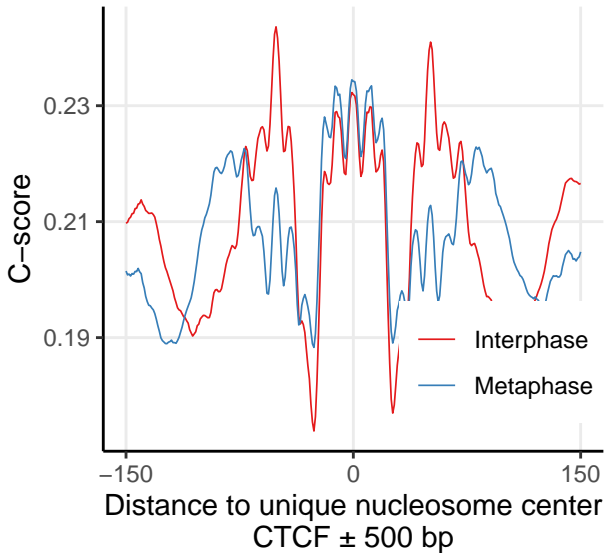

Supplement: Supplementary file 9 — Source data Fig. 7 [file 44320_2026_192_MOESM9_ESM.zip › Figure 7/7E/Figure7E.pdf]

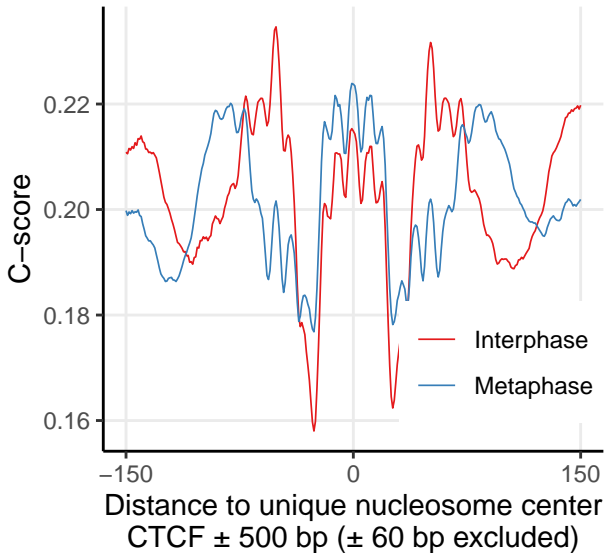

Supplement: Supplementary file 9 — Source data Fig. 7 [file 44320_2026_192_MOESM9_ESM.zip › Figure 7/7F/Figure7F.pdf]

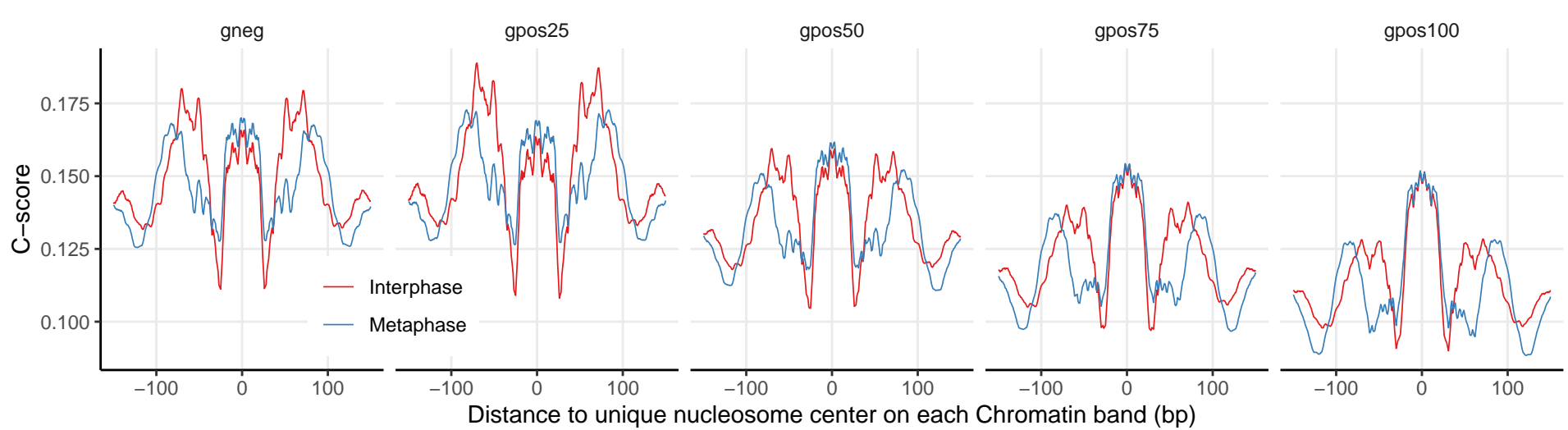

Supplement: Supplementary file 9 — Source data Fig. 7 [file 44320_2026_192_MOESM9_ESM.zip › Figure 7/7G/Figure7G.pdf]
