# Supplementary material for: Differential nucleosome organization in human interphase and metaphase chromosomes
Source: Mol Syst Biol. 2026 Feb 2;22(5):738–65. doi: 10.1038/s44320-026-00192-y (PMC13144420; doi:10.1038/s44320-026-00192-y)
Supplement: Supplementary file 6 — Source data Fig. 4 [file 44320_2026_192_MOESM6_ESM.zip › Figure 4/4D/Figure4D.pdf]

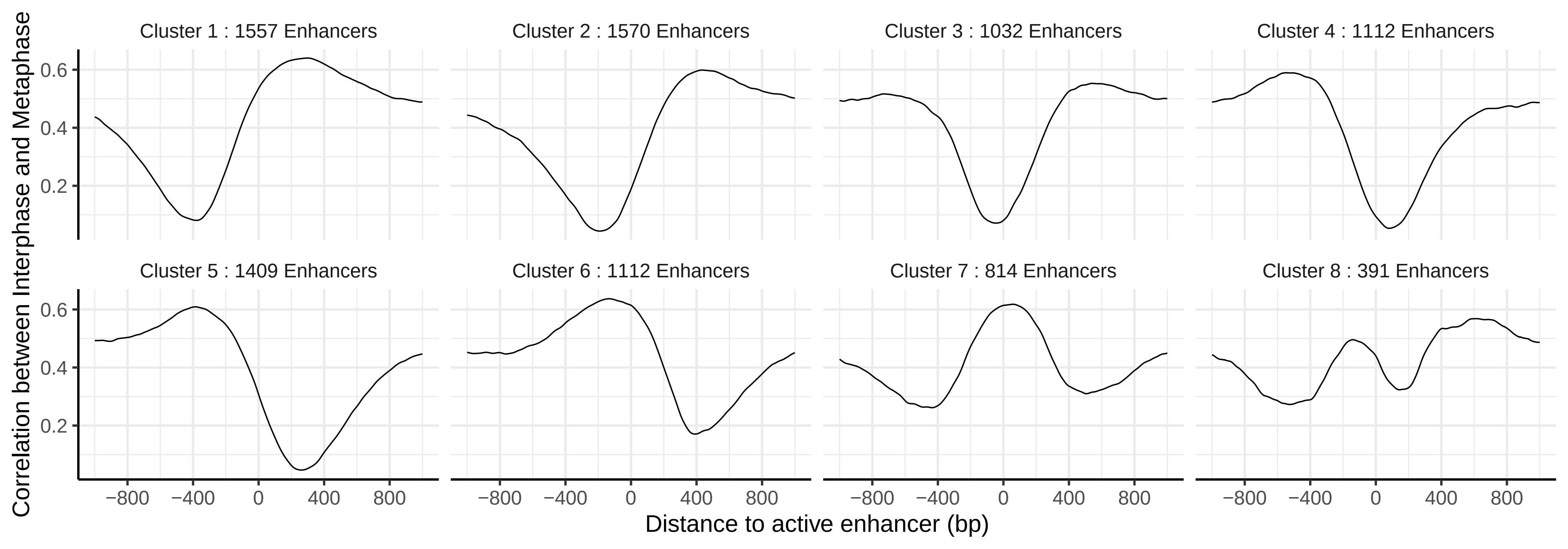
Cluster 1 : 1557 Enhancers
Cluster 2 : 1570 Enhancers
Cluster 3 : 1032 Enhancers
Cluster 4 : 1112 Enhancers
Cluster 5 : 1409 Enhancers
Cluster 6 : 1112 Enhancers
Cluster 7 : 814 Enhancers
Cluster 8 : 391 Enhancers
Correlation between Interphase and Metaphase
0.2
0.4
0.6
−800
−400
0
400
800
Distance to active enhancer (bp)
