# Supplementary material for: Differential nucleosome organization in human interphase and metaphase chromosomes
Source: Mol Syst Biol. 2026 Feb 2;22(5):738–65. doi: 10.1038/s44320-026-00192-y (PMC13144420; doi:10.1038/s44320-026-00192-y)
Supplement: Supplementary file 10 — Expanded View Figures [file 44320_2026_192_MOESM10_ESM.pdf]

Expanded View Figures

Figure EV1. Development of chemical nucleosome mapping in HeLa S3 cells.

(**A**) Design of a common RNAi target sequence shared by all human histone H4 isoforms. The siRNA antisense strand is designed to bind H4 mRNA via Watson–Crick or wobble base pairing. (**B**) Comparison of histone levels in parental and H4S47C-expressing HeLa S3 cell lines. Top: SDS-PAGE analysis of total histones purified from parental, clone 1-2, and clone 2 cells. Bottom: Western blot analysis of H4 levels in the same histone samples. (**C**) Growth curve showing that H4S47C-expressing HeLa S3 cells proliferate at the same rate as wild-type (WT) cells. Data represent the mean of biological triplicates; error bars indicate SEM. (**D**) Scatter plot of $\log_2$(FPKM) values comparing transcriptomes of clone 1-2 and clone 2. (**E**) FACScan analysis of cell cycle distribution in asynchronous (interphase) and synchronized prometaphase HeLa S3 cells. (**F**) Western blot analysis of H4 levels in total cell lysates from interphase and metaphase HeLa S3 cell lines. (**G**) DNA laddering pattern from chemically cleaved nucleosomes in interphase and metaphase clone 2 cells.

© The Author(s)

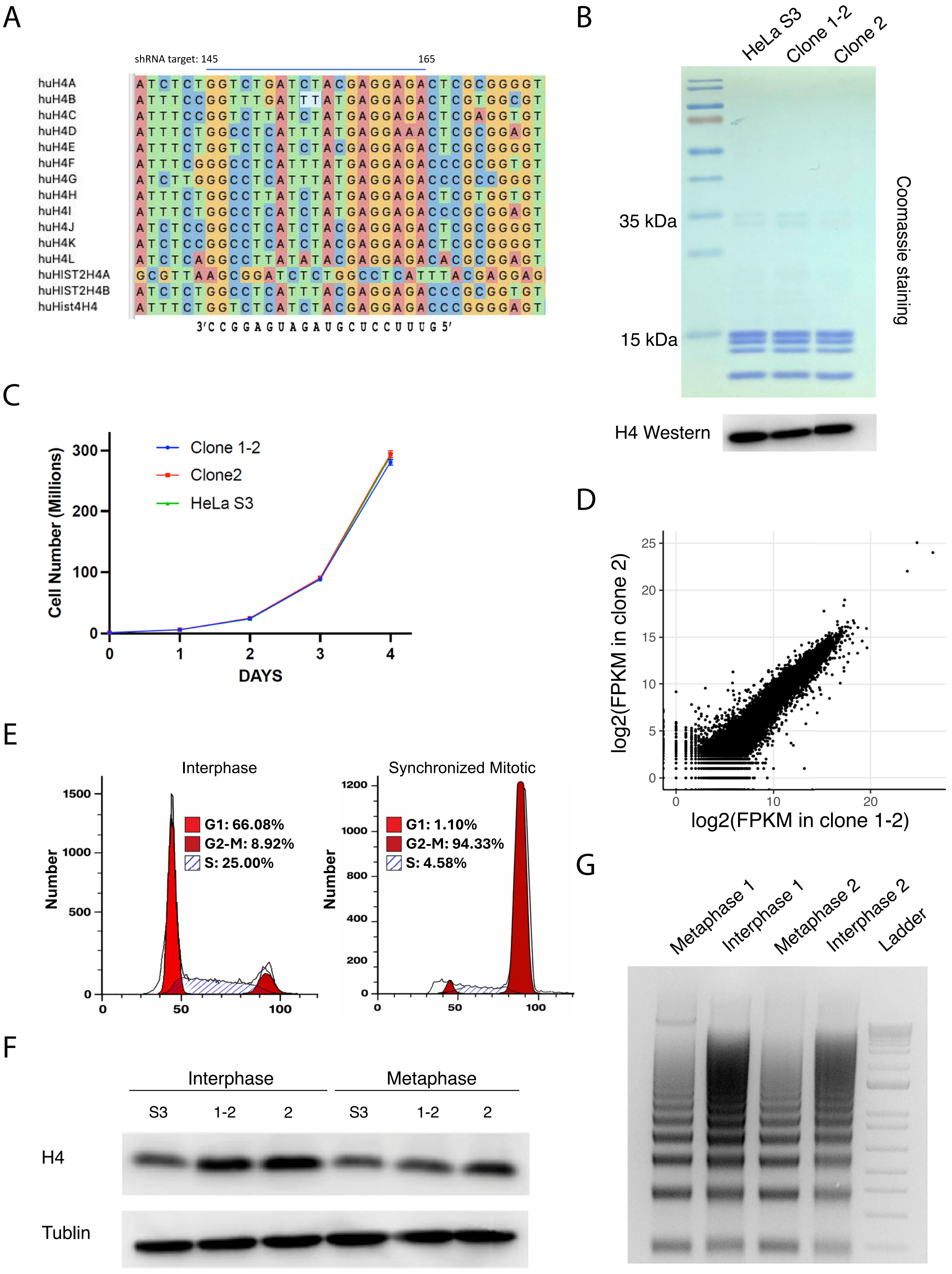
A
shRNA target: 145
165
huH4A ATCTCTGGTCTGATCTACGAGGAGACTCGCGGGGT
huH4B ATTTCCGGTTTGATTTATGAGGAGACTCGTGGCGT
huH4C ATTTCCGGTCTTATCTATGAGGAGACTCGAGGTGT
huH4D ATTTCTGGCCTCATTTATGAGGAAACTCGCGGAGT
huH4E ATTTCTGGTCTCATCTACGAGGAGACTCGCGGGGT
huH4F ATTTCGGGCCTCATTTATGAGGAGACCCGCGGTGT
huH4G ATCTTGGGCCTCATTTATGAGGAGACCCGCCGGGT
huH4H ATTTCTGGCCTTATCTATGAGGAGACTCGTGGTGT
huH4I ATTTCTGGCCTCATCTATGAGGAGACCCGCGGAGT
huH4J ATCTCCGGCCTCATCTACGAGGAGACTCGCGGGGT
huH4K ATCTCCGGCCTCATCTACGAGGAGACTCGCGGGGT
huH4L ATCTCAGGCCTTATATACGAGGAGACACGCGGAGT
huHIST2H4A GCGTTAAGCGGATCTCTGGCCTCATTTACGAGGAG
huHIST2H4B ATCTCTGGCCTCATTTACGAGGAGACCCGCGGTGT
huHist4H4 ATTTCTGGTCTCATCTACGAGGAGACCCGGGGAGT
3′C C G G A G U A G A U G C U C C U U U G 5′
B
HeLa S3
Clone 1-2
Clone 2
35 kDa
15 kDa
Coomassie staining
H4 Western
C
Cell Number (Millions)
300
200
100
0
Clone 1-2
Clone2
HeLa S3
0
1
2
3
4
DAYS
D
log2(FPKM in clone 2)
25
20
15
10
5
0
10
20
log2(FPKM in clone 1-2)
E
Interphase
Number
1500
1000
500
0
50
100
G1: 66.08%
G2-M: 8.92%
S: 25.00%
Synchronized Mitotic
Number
1200
1000
800
400
200
0
50
100
G1: 1.10%
G2-M: 94.33%
S: 4.58%
F
Interphase
Metaphase
S3
1-2
2
S3
1-2
2
H4
Tublin
G
Metaphase 1
Interphase 1
Metaphase 2
Interphase 2
Ladder

 © The Author(s)

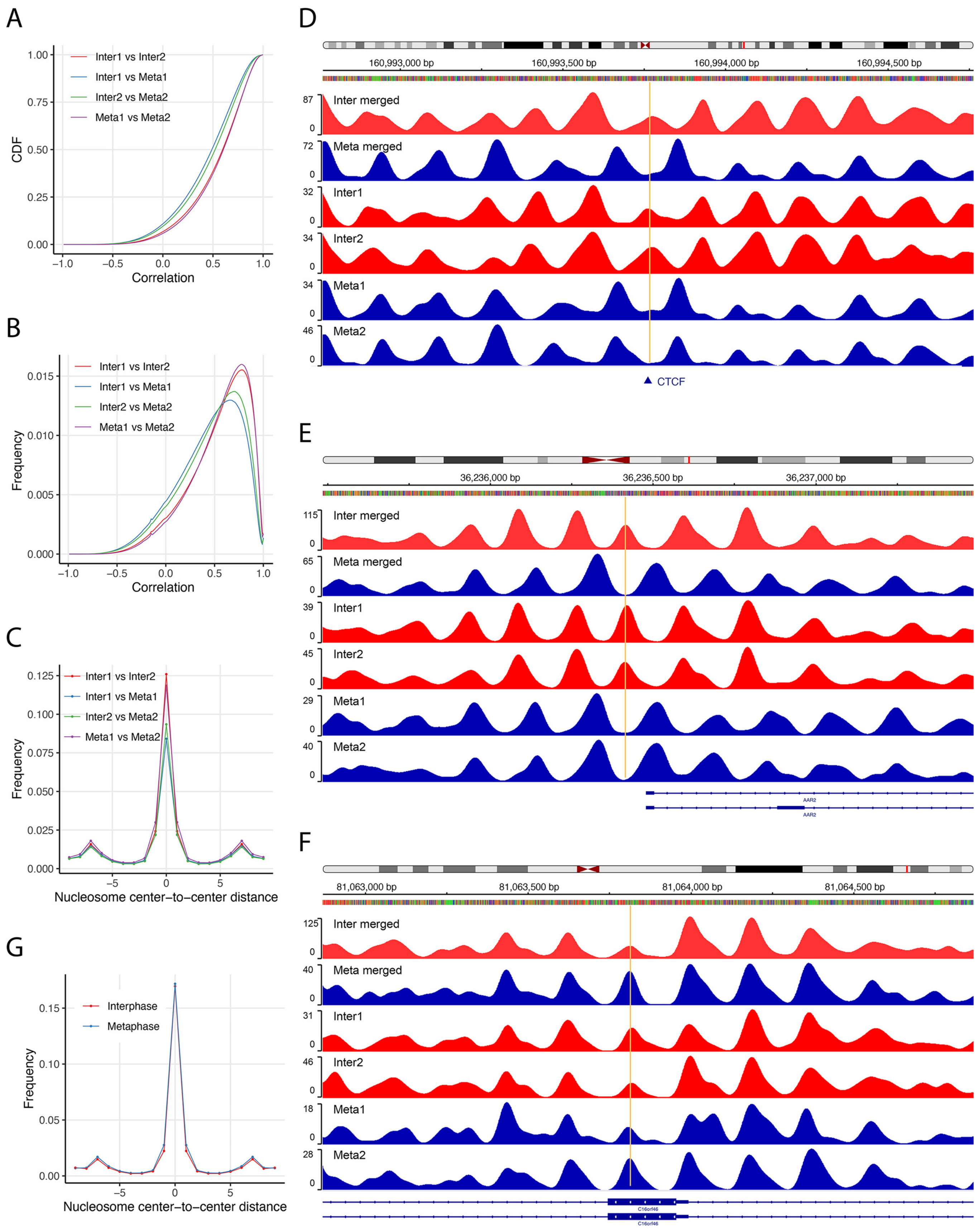
A
CDF
Inter1 vs Inter2
Inter1 vs Meta1
Inter2 vs Meta2
Meta1 vs Meta2
Correlation
B
Frequency
Correlation
C
Frequency
Nucleosome center–to–center distance
G
Interphase
Metaphase
Frequency
Nucleosome center–to–center distance
D
160,993,000 bp
160,993,500 bp
160,994,000 bp
160,994,500 bp
Inter merged
Meta merged
Inter1
Inter2
Meta1
Meta2
CTCF
E
36,236,000 bp
36,236,500 bp
36,237,000 bp
AAR2
F
81,063,000 bp
81,063,500 bp
81,064,000 bp
81,064,500 bp
C16orf46

Figure EV2. Reproducibility of chemical nucleosome maps in H4S47C-expressing HeLa S3 cells.

(**A**) Empirical cumulative distribution function (ECDF) plots showing genome-wide local Pearson correlation of nucleosome occupancy scores between biological replicates of clone-2 H4S47C-expressing cells. Comparisons include: Interphase 1 vs. Interphase 2, Metaphase 1 vs. Metaphase 2, Interphase 1 vs. Metaphase 1, and Interphase 2 vs. Metaphase 2. Correlations were computed using a 501-bp sliding window with 1-bp step size. (**B**) Frequency distribution of local correlation values for the same replicate comparisons shown in (**A**). (**C**) Distribution of center-to-center distances between unique nucleosomes identified in each pairwise comparison from (**A**). (**D–F**) Representative genomic loci illustrating nucleosome occupancy scores before and after merging clone-2 biological replicates. (**G**) Frequency plot of center-to-center distances between unique nucleosomes in independent clones. Shown are comparisons between clone 1-2 and clone 2 for both interphase and metaphase chemical nucleosome maps.

 © The Author(s)

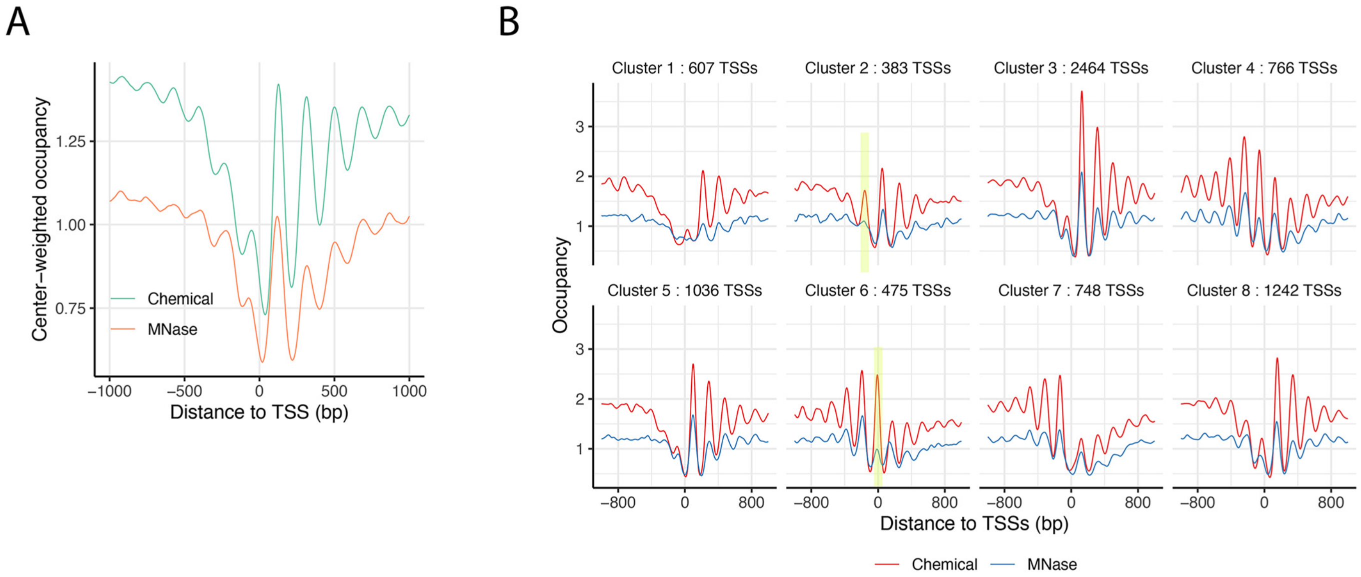

Figure EV3. Nucleosome occupancy over TSSs during interphase.

(**A**) Average interphase nucleosome occupancy profiles from chemical (green) and MNase (red) maps across the TSSs of 19,166 protein-coding genes. (**B**) A subset of 7,944 TSSs, representing the top 50% of expressed genes, was grouped into eight clusters using K-means clustering based on interphase chemical occupancy scores in the region spanning –150 to +250 bp relative to the TSS (same data as Fig. 3D). Shown are the interphase occupancy profiles from the chemical and MNase maps for each cluster. Notably, clusters 2 and 6 exhibit a well-positioned –1 nucleosome in the chemical map that is not detected in the MNase map.

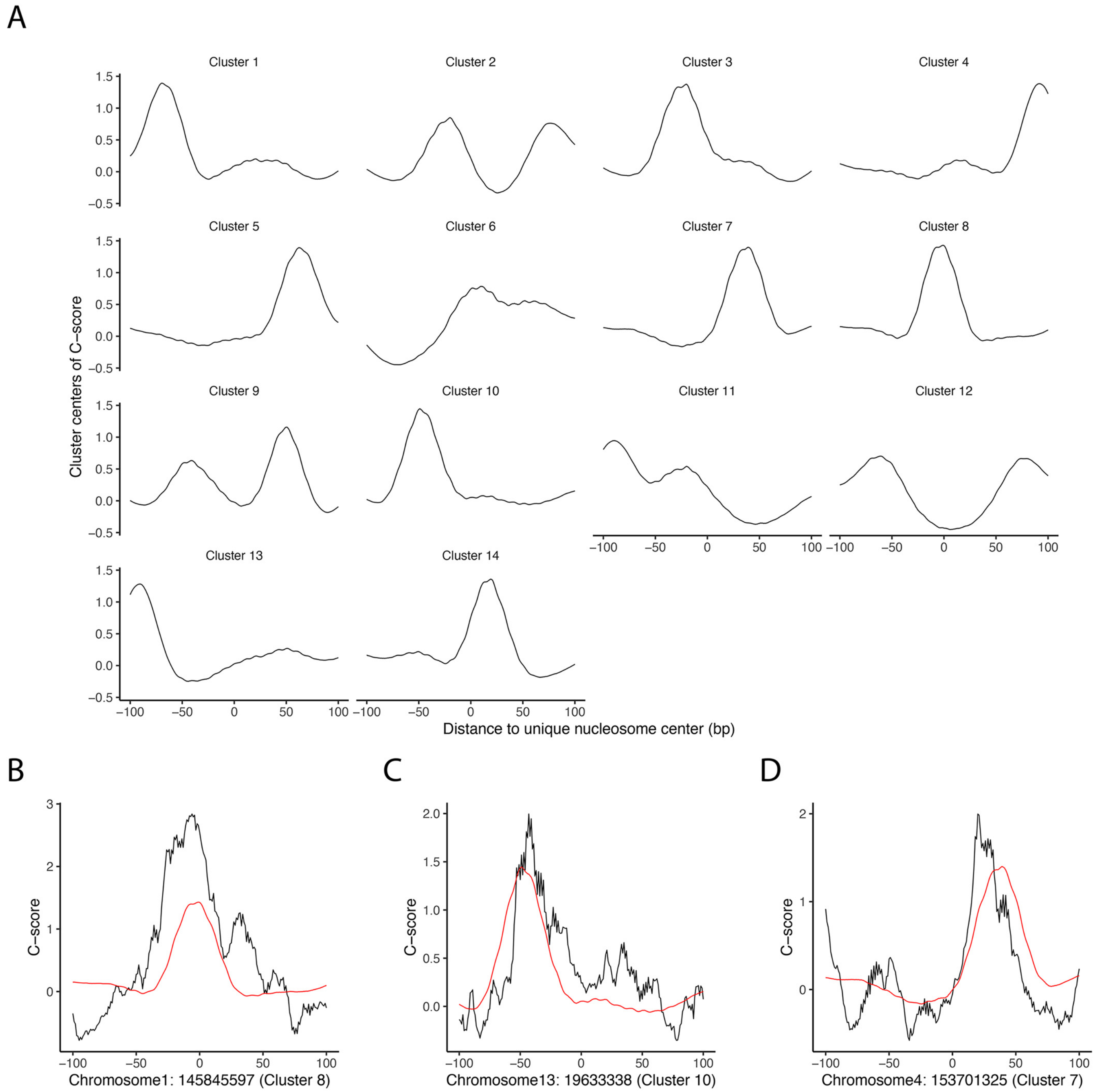

Figure EV4. Distribution of DNA C-scores within nucleosome regions.

(**A**) Unique nucleosomes near transcription start sites (TSSs) in interphase were clustered into 14 groups using a K-means algorithm, revealing distinct patterns of intrinsic DNA cyclizability (C-scores) within the nucleosome region. (**B–D**) C-score distributions for three representative nucleosomes (black). Red curves indicate the average profile for the corresponding cluster.

 © The Author(s)

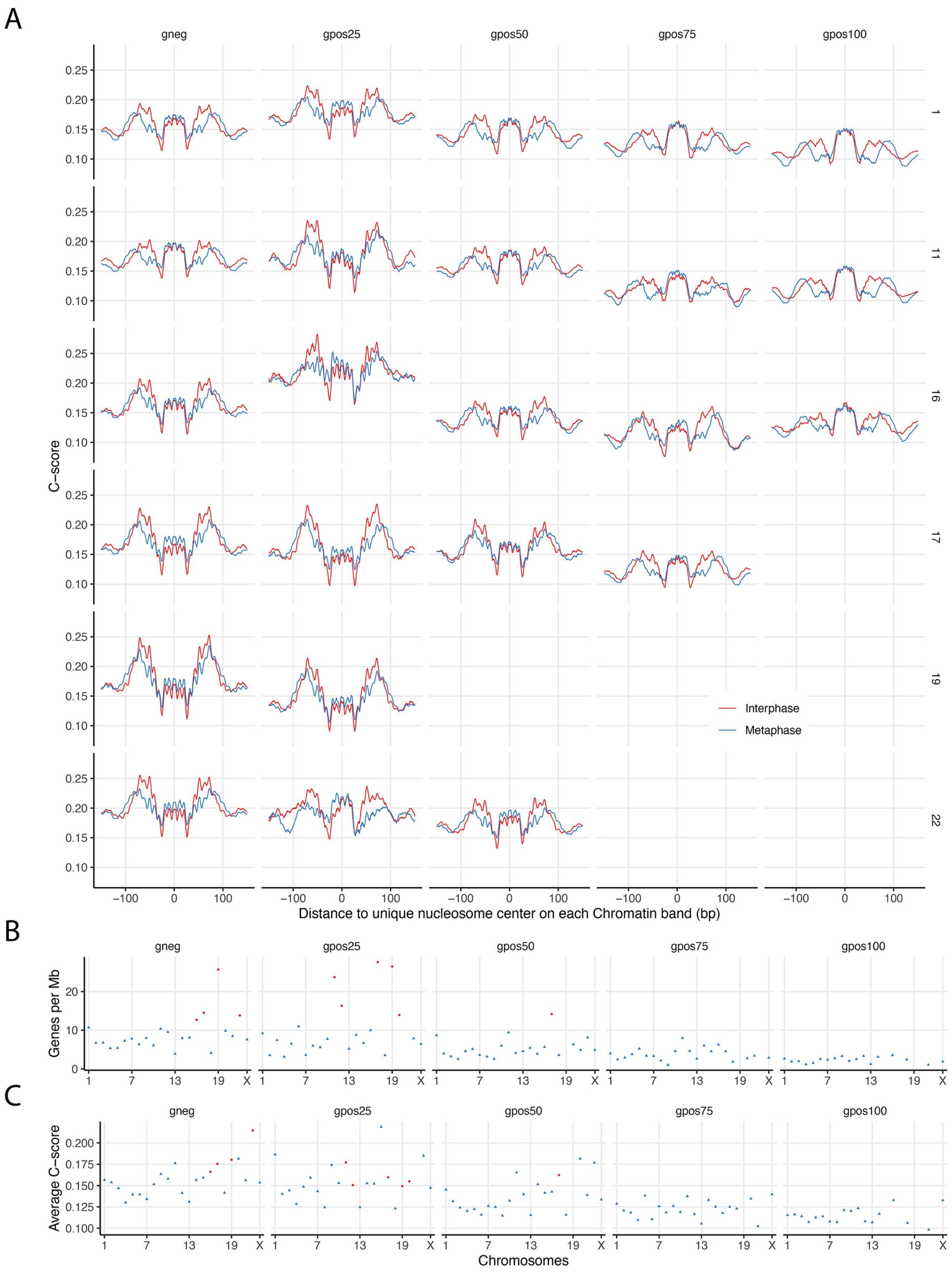
A
gneg
gpos25
gpos50
gpos75
gpos100
C-score
1
11
16
17
19
22
Interphase
Metaphase
Distance to unique nucleosome center on each Chromatin band (bp)
B
Genes per Mb
C
Average C-score
Chromosomes

◀ **Figure EV5. Relationship between gene density and nucleosomal C-score patterns.**

(**A**) Representative examples of nucleosomal C-score distributions across various G-band regions on different chromosomes. (**B**) Gene density (genes per Mb) across G-band regions on individual chromosomes. Regions with gene density >11 genes/Mb are marked as red dots; all others are shown as blue triangles. These regions correspond to those in (A) and exhibit significantly higher C-scores in the nucleosome shoulder compared to the dyad. (**C**) Distribution plot showing average C-score values within different G-band regions. Regions are colored according to gene density, as in (**B**).

 © The Author(s)
